# A Triple-Targeted Rutin-Based Self-Assembled Delivery Vector for Treating Ischemic Stroke by Vascular Normalization and Anti-Inflammation via ACE2/Ang1-7 Signaling

**DOI:** 10.1021/acscentsci.3c00377

**Published:** 2023-06-05

**Authors:** Tingkui Zhao, Fujin He, Keqing Zhao, Lin Yuxia, Huanyu Li, Xingru Liu, Juan Cen, Shaofeng Duan

**Affiliations:** † Key Laboratory of Natural Medicine and Immune Engineering, School of Pharmacy, Henan University, Kaifeng 475004, China; ‡ Institute for Innovative Drug Design and Evaluation, School of Pharmacy, Henan University, Kaifeng 475004, China; § Henan International Joint Laboratory of Chinese Medicine Efficacy, Henan University, Kaifeng 475004, China

## Abstract

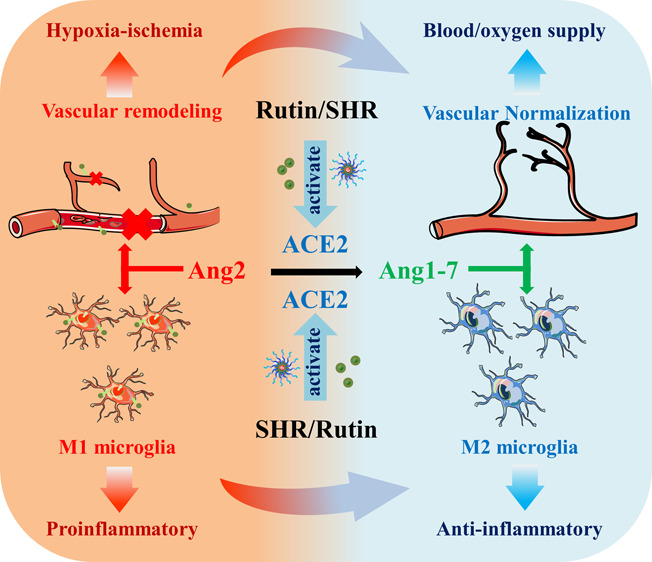

Changes in the cerebral microenvironment caused by acute ischemic stroke-reperfusion are the main obstacle to the recovery of neurological function and an important cause of stroke recurrence after thrombolytic therapy. The intracerebral microenvironment after ischemia-reperfusion reduces the neuroplasticity of the penumbra and ultimately leads to permanent neurological damage. To overcome this challenge, we developed a triple-targeted self-assembled nanodelivery system, which combines the neuroprotective drug rutin with hyaluronic acid through esterification to form a conjugate, and then connected SS-31, a small peptide that can penetrate the blood brain barrier and target mitochondria. Brain targeting, CD44-mediated endocytosis, hyaluronidase 1-mediated degradation, and the acidic environment synergistically promoted the enrichment of nanoparticles and drug release in the injured area. Results demonstrate that rutin has a high affinity for ACE2 receptors on the cell membrane and can directly activate ACE2/Ang1-7 signaling, maintain neuroinflammation, and promote penumbra angiogenesis and normal neovascularization. Importantly, this delivery system enhanced the overall plasticity of the injured area and significantly reduced neurological damage after stroke. The relevant mechanism was expounded from the aspects of behavior, histology, and molecular cytology. All results suggest that our delivery system may be an effective and safe strategy for the treatment of acute ischemic stroke-reperfusion injury.

## Abstract 2

A triple-targeted rutin-based self-assembled delivery vector treats ischemic stroke by vascular normalization and anti-inflammation via activating ACE2/Ang1-7 signaling in ischemic penumbra.

## Introduction

Stroke is one of the leading causes of severe long-term disability and death worldwide, among which acute ischemic stroke is the main stroke type in the clinic, mainly caused by sudden cerebrovascular infarction.^1^ Reperfusion therapy for patients with ischemic stroke has been developing rapidly, but the clinical prognosis of most patients is still poor despite successful restoration of blood flow.^2,3^ Angiogenesis, the growth of new blood vessels, is a natural defense mechanism that helps restore oxygen and nutrient supply to the affected brain tissue after ischemic stroke and is a key feature of ischemic stroke recovery and poststroke neuronal reorganization. Conventional wisdom holds that by stimulating blood vessel growth, angiogenesis may stabilize cerebral perfusion, thereby promoting neuronal survival, brain plasticity, and neural recovery.^4,5^ However, there is increasing evidence that both compensatory brain microvessels after stroke and those generated by direct stimulation of angiogenic factors such as VEGF are more permeable than normal vessels and are prone to rupture, which can induce a large amount of reactive oxygen species (ROS) and cause vascular neuroinflammation and vascular dysfunction, thus becoming the main factor of reperfusion injury.^6−8^ This also explains why the highly valued proangiogenic therapies have always been unsatisfactory in the clinic. To date, stabilizing the structure of new blood vessels and promoting vascular normalization have received increasing attention in the treatment of solid tumors,^9,10^ but less research has been done on ischemic stroke.

The central renin angiotensin system (RAS) plays an important role in maintaining the homeostasis of vascular function. On the one hand, angiotensin II (Ang2), the main active substance of RAS, is significantly up-regulated in stroke tissue.^11^ Excessive Ang2 can lead to the increase of ROS, structural damage, and dysfunction in vascular endothelial cells, thereby promoting the activation of M1 microglia and the release of various inflammatory mediators.^12^ It has been reported that capillary injury caused by cerebral ischemic attack can cause the adhesion and aggregation of leukocytes and platelets, thereby triggering the formation of secondary thrombosis and affecting the therapeutic effect of thrombolysis.^13^ On the other hand, angiotensin converting enzyme 2 (ACE2), widely regarded as the receptor of COVID-19,^14^ is a key regulator of the renal angiotensin system, whose main function is to catalyze the production of Ang1-7 from Ang2,^15^ As a physiological antagonist of Ang2, Ang1-7 can inhibit the apoptosis of endothelial cells, stabilize the structure of vascular endothelial cells, promote vascular normalization, inhibit the excessive inflammatory response caused by ACE/Ang2, and polarize microglia from M1 phenotype to M2 phenotype.^16−18^ All this indicates that ACE2 can be used as a potential powerful target for the treatment of ischemic stroke. Rutin (RT) is a kind of natural flavonoid glycoside with pharmacological effects such as anti-inflammatory, antioxidant, and enhancing vascular toughness.^19^ Recent studies have shown that rutin has a strong affinity for ACE2 and can activate ACE2/Ang1-7 to play a protective role.^20,21^ However, its low water solubility and difficulty in crossing the blood brain barrier (BBB) greatly limit its clinical application.

In this study, a polymeric micelle system (SS-31-hyaluronic acid-rutin, designated as SHR) with brain targeting and ischemic penumbra enrichment was constructed. Notably, SS-31 is a synthetic mitochondria-targeting peptide that can freely penetrate the cell membrane and concentrate on the inner membrane of mitochondria in an energy-independent and unsaturated form.^22^ Hyaluronic acid (HA) is an ideal drug carrier with high hydrophilicity, high viscoelasticity, biodegradability, low sensitization, good biocompatibility, and the ability to bind to specific receptors on the cell surface.^23^ In addition, HA can independently form redox-sensitive micelles in aqueous solution by combining with curcumin through a disulfide-containing linker, which improves the water solubility of the drug.^24^ Accumulating evidence suggests that SS-31 can easily cross the BBB and exerts certain neuroprotective effects due to its aromatic and cationic properties.^25,26^ Nevertheless, its antioxidant effect is negligible in the treatment of cerebral ischemia when its amount is very small. Herein, we merely grafted it as a targeted peptide onto HA-RT micelles (designated HR) to enhance the ability of micelles to cross the BBB regardless of its protective effect. Then, with the help of the acidic environment caused by ischemia and hypoxia, as well as the overexpression of CD44 and hyaluronidase 1, drugs can achieve targeted enrichment and sustained release in the ischemic area of the brain. The results of in vivo and in vitro experiments demonstrate that the SHR micelles can be effectively enriched in the ischemic area of the brain and exerte ideal curative effects on ischemic stroke through antioxidation and anti-inflammatory effects, and promotion of survival and normalization of new blood vessels.

## Results and Discussion

### Fabrication and Physicochemical Characterization of SHR Micelle

**Figure 1:**
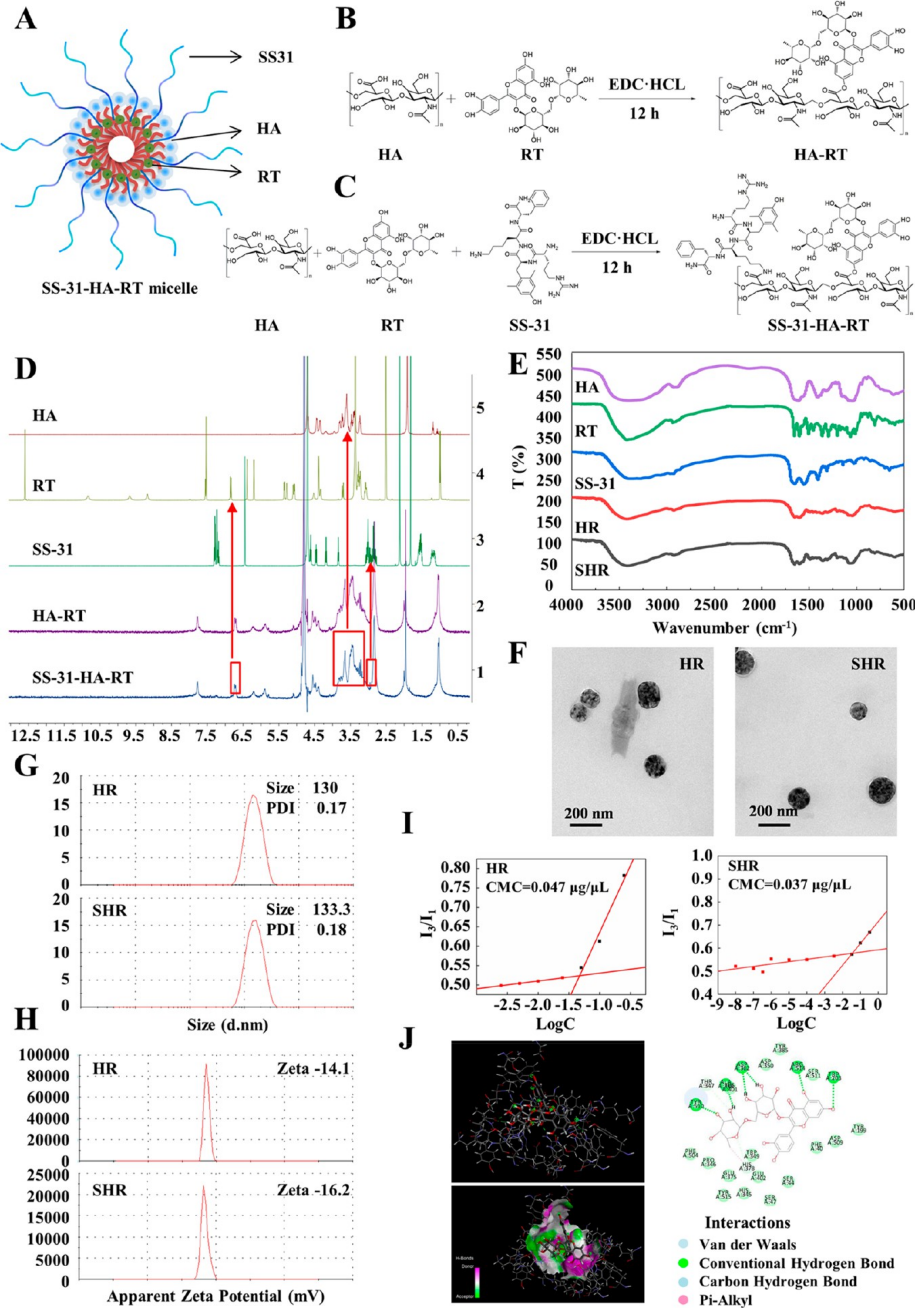
Synthesis and characterization of SHR micelles. (A) Schematic diagram of SHR micelles. (B) Synthetic route of HA-RT micelles. (C) Synthetic route of SHR micelles. (D) ^1^H NMR spectra of HA, RT, SS-31, HA-RT, and SS-31-HA-RT. (E) Circular dichroism spectra of HA, RT, SS-31, HA-RT, and SS-31-HA-RT. (F) Representative TEM images of the morphology of HR and SHR micelles. (Scale bar = 200 nm.) (G) Particle sizes of HR micelles and SHR micelles. (H) Zeta potentials of HR micelles and SHR micelles. (I) Critical micelle concentrations (CMCs) of HR micelles and SHR micelles. (J) Molecular docking of RT and ACE2.

In this study, a triple-targeting micelle (SHR) was developed based on SS-31-modified HR conjugates. SHR micelles (Figure 1A) were fabricated as follows: (i) HA, a glycosaminoglycan composed of disaccharides with d-glucuronic acid and *n*-acetylglucosamine as repeating units, was dissolved in ddH_2_O to form a solution which was cooled to 0 °C, followed by the addition of the activating agent EDC·HCl, and followed by the addition of a solution of RT in DMF. The reaction proceeded at room temperature in the dark overnight, and then the mixture solution was transferred into a dialysis tubing (MWCO 3500) to be dialyzed against ddH_2_O for 12 h followed by lyophilization to afford HA-RT as a white powder; (ii) the lyophilized powder, HA-RT, was dissolved in ddH_2_O to form a solution which was cooled to 0 °C, followed by the addition of the activating agent EDC·HCl, and followed by the addition of a solution of a mitochondria-targeting peptide, 2,6-dimethyl-l-tyrosine (SS-31), in ddH_2_O. The reaction proceeded at room temperature in the dark overnight, and then the mixture solution was transferred into a dialysis tubing (MWCO 3500) to be dialyzed against ddH_2_O for 12 h and then lyophilized to afford SHR (Figure 1B,C). The solubility test showed that the solubility of RT was 0.194 mg/mL, the solubility of HR was 769 mg/mL, and the solubility of SHR was 725 mg/mL, suggesting that the solubility of RT was increased about 4000 times through optimization. Then, the SHR micelles were characterized by ^1^H NMR (Figure 1D) and FT-IR (Figure 1E) spectroscopy. The morphology of HR and SHR was observed under transmission electron microscopy (TEM) after negative staining with 2% phosphotungstic acid solution. The TEM results showed that both HR and SHR were homogeneous spherical particles (Figure 1F). The particle sizes of HA-RT and SHR were 130 and 133.3 nm, respectively, and their zeta potentials were −14.1 mV and −16.2 mV, respectively (Figure 1G,H). The pyrene fluorescence probe spectrometry results demonstrate that the critical micelle concentrations (CMCs) of HR micelles and SHR micelles were 0.047 μg/μL and 0.037 μg/μL, respectively (Figure 1I). In addition, molecular docking studies showed that the CDOCKER energy value is 7.95964, while there are 6 hydrogen bonds with strong affinity (Figure 1J). All these results indicate that RT has a high affinity for ACE2 protein, which is in accordance with reported studies.^20,21^

### SHR Showed a Triple-Targeting Effect: Penetration across the BBB, Enrichment in the Ischemic Area, and Located in the Mitochondria

**Figure 2:**
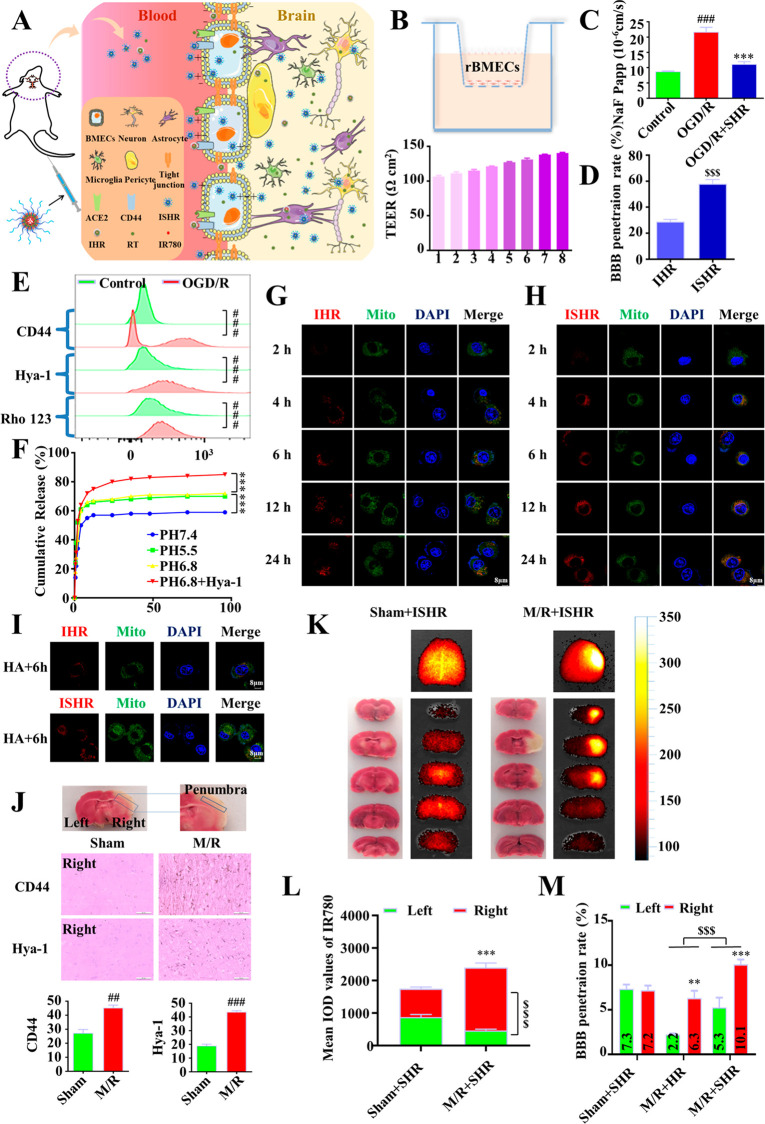
Targeting, release, and distribution of SHR micelles in the brain tissue. (A) Schematic diagram of SHR micelles crossing blood brain barrier. (B) Establishment of blood-brain barrier model and determination of TEER in vitro. (C) Apparent permeability coefficient (Papp) of NaF was calculated to reflect the integrity of the blood-brain barrier. (D) Changes in the ability of micelles to cross the blood-brain barrier in vitro before and after connecting SS-31. (E) Two hours after OGD injury, the expression of CD44, Hya-1 and the accumulation of Rho 123 in SH-SY5Y cells were detected by flow cytometry. (F) Release of SS-31-HA-RT micelles in buffers of pH 5.5, 6.8, 7.4, and 6.8 with Hya-1, respectively. (G) Uptake and distribution of HA-RT micelles in SH-SY5Y cells with oxygen-glucose deprivation-injury. (H) Uptake and distribution of SHR micelles in SH-SY5Y cells with oxygen-glucose deprivation-injury. (I) Effect of free hyaluronic acid pretreatment on cellular uptake of HR micelles and SHR micelles. (J) Expression of CD44 and hyaluronidase-1 in the penumbra of cerebral ischemia in rats. (K) Ex vivo IVIS imaging of brain and brain slices from sham or tMCAO rat models 24 h after i.v. injection of SHR micelles carrying IR780. (L) Fluorescence quantitative statistics of (K). (M) Quantified distribution of HR and SHR in the ischemia-affected hemisphere (right) and contralateral nonischemic hemisphere (left) by UV. Data were expressed by mean ± SEM (*n* = 3), ^#^*P* < 0.05, ^##^*P* < 0.01, ^###^*P* < 0.001 compared with the control group; *P < 0.05, **P < 0.01, ***P < 0.001 compared with the model group; ^$^*P* < 0.05, ^$$^*P* < 0.01, ^$$$^*P* < 0.001 compared with the HR group; ^@^*P* < 0.05, ^@@^*P* < 0.01, ^@@@^*P* < 0.001 compared with the nonischemic hemisphere group.

A schematic diagram of the triple targeting of SHR is shown in Figure 2A. The drug pathway in vivo was investigated by incubating the conjugate-based micelles with the fluorescent molecule IR780 (IHR and ISHR). First, in order to evaluate the efficiency of SHR micelles crossing the BBB, we established an in vitro BBB model (Figure 2B). After the transmembrane resistance was stabilized, the changes of BBB permeability (NaF) and drug accumulation in the lower chamber were measured before and after the administration. The results showed that the efficiency of the micelles penetrating the BBB model in vitro was nearly doubled after SS-31 was connected, and the permeability damage of the BBB was repaired by the drug (Figure 2C,D), implying that the enhanced BBB penetrating ability could be attributed to SS-31, an artificial mitochondria-targeting peptide that can be concentrated on the inner mitochondrial membrane in an energy-independent and unsaturated form. This peptide contains an alternating aromatic-cationic motif that allows it to freely permeate through the cell membrane.^27^ Importantly, the uptake of SS31 by mitochondria is independent of the mitochondrial transmembrane potential, indicating that this short peptide also accumulates in injured cells, which is very useful for treating damaged tissues. To our best knowledge, very little research has been conducted on the ability of SS-31 to penetrate the BBB so far. Our group has carried out the related studies and verified that SS-31 can effectively penetrate the BBB and distribute rapidly, which may be due to its surface charge density, lipid binding density, and affinity.^28,29^

Second, the enrichment of SHR in the ischemic area was analyzed. The results demonstrate that after treatment with oxygen glucose deprivation (OGD), the expression of CD44 and hyaluronidase 1 (Hya-1) and the content of rhodamine 123 in SH-SY5Y cells were significantly increased (Figure 2E). Accordingly, the results of brain slices of transient middle cerebral artery occlusion (tMCAO) rats also showed that CD44 and Hya-1 were overexpressed in the injured tissue (Figure 2J). Therefore, it can be concluded that, with the help of SS-31 and HA, SHR can be enriched into the ischemic tissue. As the redox-sensitive micelles SHR micelles are easily disrupted by the acidic environment formed by the accumulation of CO_2_ from lactate and anaerobic glycolysis, and Hya-1, RT can be released sustained from the micelles in the injured area (Figure 2F). In addition, we performed small animal imaging 24 h after intravenous injection of IHR and ISHR micelles to assess the biodistribution. As shown in Figure 2K, the amount of drug crossed the BBB by SS-31-conjugated micelles is much greater than that without SS-31 (Figure 2L). Detection of the drugs extracted from the brain tissue by UV assays also showed that, with the assistance of SS-31, the amount of drug that crossed the BBB was doubled (Figure 2M).

Third, compared with IHR, the rapid uptake of ISHR was more prone to converge to mitochondria, which is also the result of the connection to SS-31 (Figures 2G,H and S1). After pretreatment with free HA, the uptake of HR micelles was significantly inhibited, while the ability of SHR micelles to enter cells was almost unaffected (Figure 2I), indicating that SHR micelles can enter cells independently of CD44-mediated endocytosis. Under the influence of the factors mentioned above, the SHR micelles enriched in the ischemic area were mainly transformed into three forms: SHR micelles, HR micelles, and free RT. SHR micelles with intact morphology either enter cells via CD44 receptor-mediated endocytosis or enter into cells and target damaged mitochondria through the excellent transmembrane action of SS-31. However, HR micelles, which were generated by partial degradation of HA by Hya-1,^30^ can also enter cells via CD44 receptor-mediated endocytosis. Free RT, which was released by SHR or HR micelles, can directly bind to ACE2 receptors on the surface of the cell membrane to exert therapeutic effects on the damaged tissue. Taken together, all these in vitro and in vivo results confirm that the drug delivery system has the advantages of effectively crossing the blood-brain barrier, enriching in the injured brain area and targeting drug delivery to subcellular organelles’ mitochondria.

### SHR Micelles Showed Great Therapeutic Effects on Zebrafish Models of Inflammatory, Oxidative Stress, Angiogenesis Inhibition, and Neuromania

**Figure 3:**
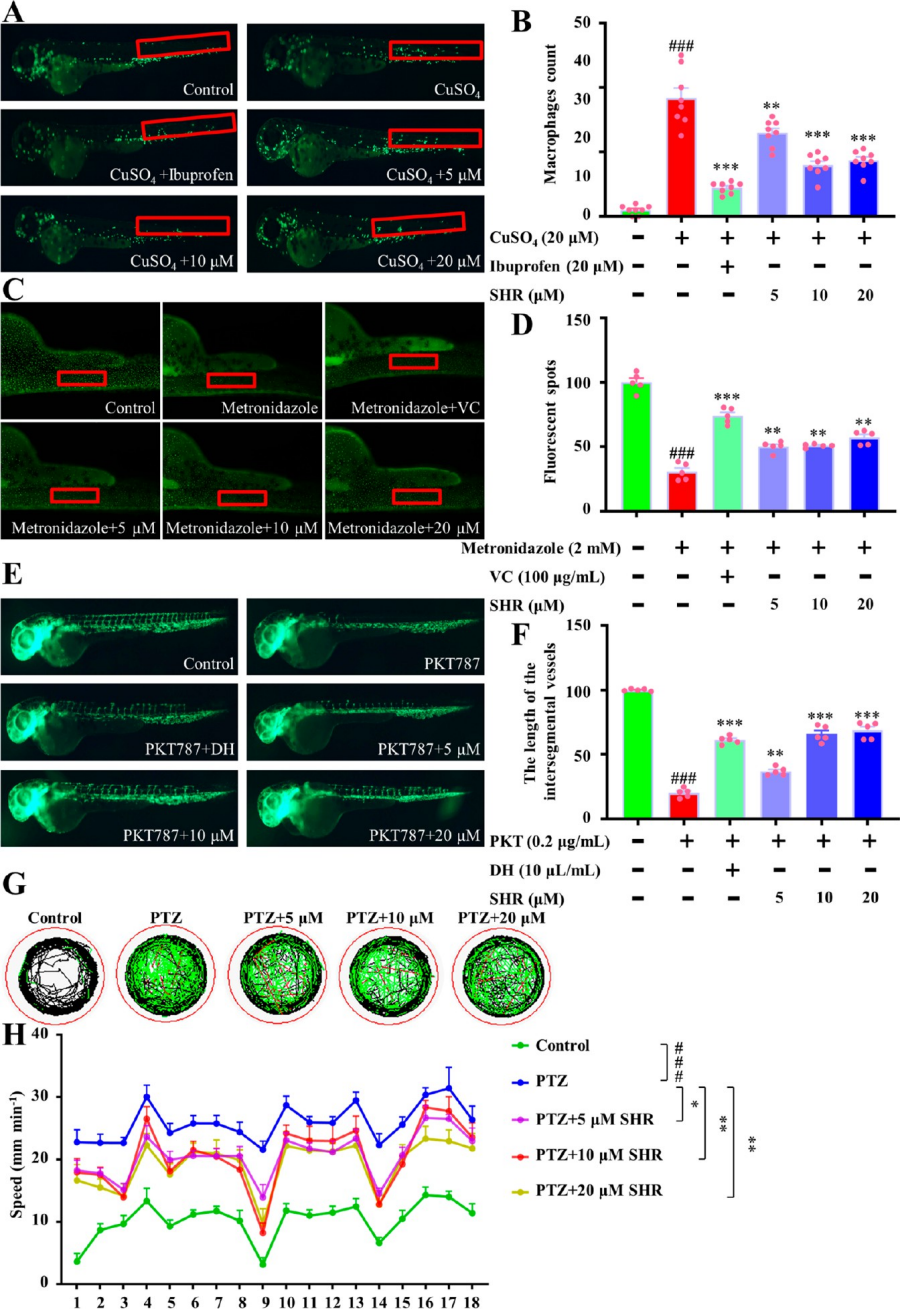
Protective effects of SHR against inflammation, oxidation, antiangiogenesis, and neuromania in zebrafish models. (A) Anti-inflammatory effects of SHR micelles on the zebrafish model of inflammation. (B) Quantitative statistics of anti-inflammatory effects of SHR on the zebrafish model of inflammation. (C) Antioxidant effects of SHR micelles on the zebrafish model of oxidative stress. (D) Quantitative statistics of antioxidant effects of SHR on the zebrafish model of oxidative stress. (E) Pro-angiogenic effect of SHR micelles on the zebrafish model of angiogenesis inhibition. (F) Quantitative statistics of the pro-angiogenic effect of SHR on the zebrafish model of angiogenesis inhibition. (G) Neuroprotective effect of SHR micelles on the zebrafish model of neuromania. (H) Quantitative statistics of the neuroprotective effect of SHR on the zebrafish model of neuromania. Data were expressed by mean ± SEM (*n* = 5–8). ^*#*^*P* < 0.05, ^*##*^*P* < 0.01, ^*###*^*P* < 0.001 compared with the control group; ^*^P < 0.05, ^**^P < 0.01, ^***^P < 0.001 compared with the model group.

In order to prescreen the effects of SS31-HA-RT micelles in vivo, we established zebrafish models of inflammatory, oxidative stress, angiogenesis inhibition, and neuromania. As shown in Figure 3A,B, treatment of juvenile zebrafish with 20 μM CuSO_4_ significantly increased the number of macrophages around the nerve column, resulting in an inflammatory response, while ibuprofen significantly reduced the accumulation of macrophages induced by CuSO_4_ and the number of macrophages around the nerve column. Likewise, different concentrations of SS31-HA-RT micelles also significantly reduced the number of macrophages around the nerve column, suggesting that the SHR group had the same anti-inflammatory effect as the ibuprofen group.

Metronidazole (MTZ) treatment of zebrafish can effectively remove the specific cells of transgenic zebrafish expressing nitrogen reductase (NTR) and rapidly produce reactive oxygen species (ROS) in specific tissues or cells. Therefore, MTZ-treated Tg (krt4: NTR-hKikGR) cy17 zebrafish were used as a model for a ROS assay. As shown in Figure 3C,D, compared with the model group, SS31-HA-RT micelles at concentrations of 5, 10, and 20 μM significantly decreased the fluorescence points of zebrafish skin, suggesting that SS31-HA-RT micelles had a good antioxidant effect similar to vitamin C.

As shown in Figure 3E,F, the model drug (0.2 μg/mL PKT787) had a significant inhibitory effect on the intersegmental vessels of zebrafish, while ibuprofen (10 μL/mL DH) had significant angiogenic activity. Here, it is worth noting that different concentrations of SHR also showed significant angiogenic activity in the presence of PKT787.

In the neuroprotective experiment, PTZ (15 mM) caused mania in zebrafish, which was characterized by increased locomotion. Compared with the model group, different concentrations of SHR significantly inhibited PTZ-induced acceleration of movement ability, suggesting that SHR has a potential neuroprotective effect on neurological abnormality (Figure 3G,H).

### SHR Micelles Restored Injured Neurons and Behavioral Functions in Rats with Cerebral Ischemia and Reperfusion (I/R)

**Figure 4:**
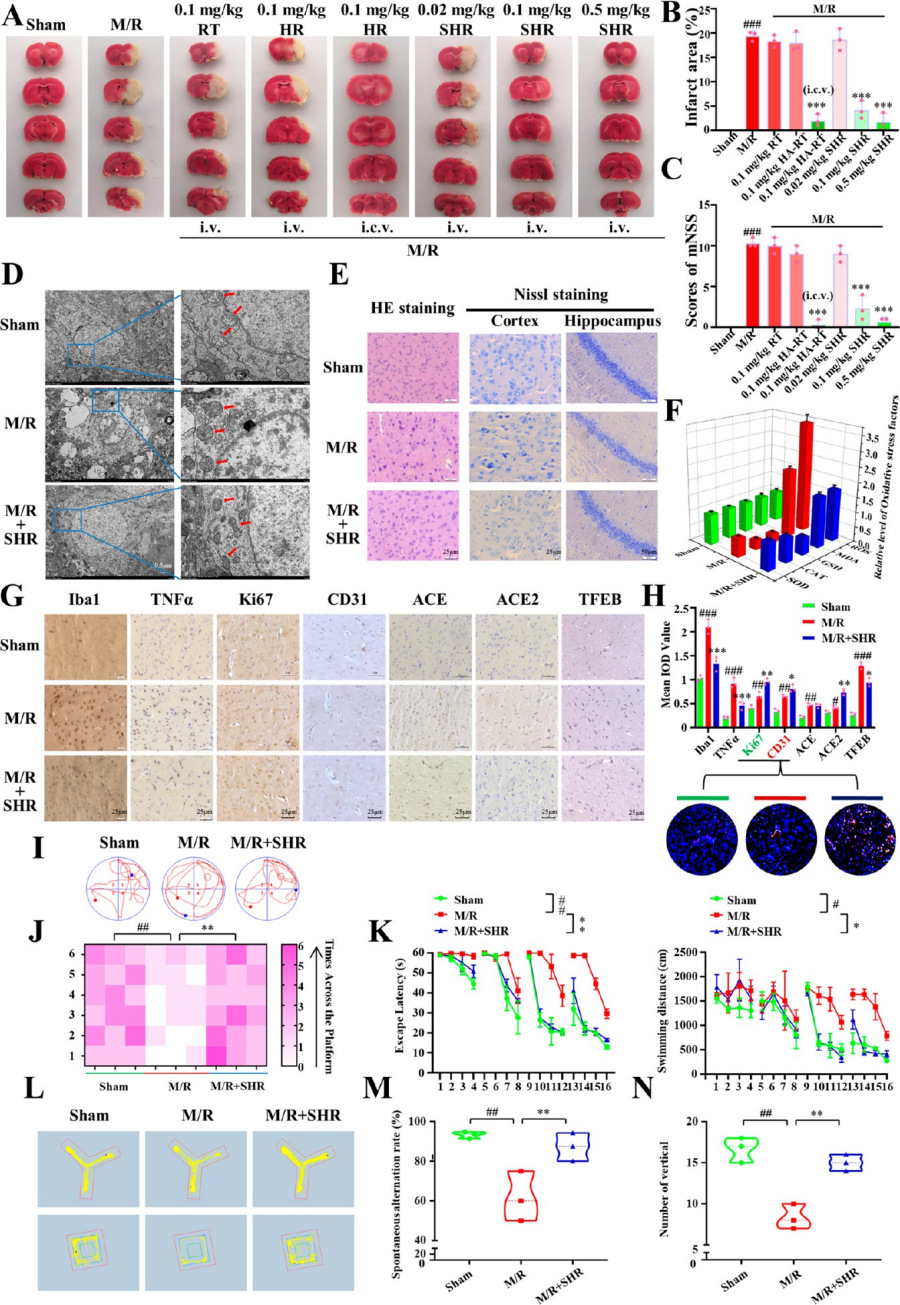
In vivo neuroprotective effect of SHR micelles in tMCAO rats. (A) Representative TTC staining images of brain slices treated with different formulations. (B) Quantification of the infarct area of TTC staining. (C) mNSS neurological deficit score of each group. (D) Representative electron microscopy images of ischemic penumbra cells (arrow, mitochondria; scale bar = 5 and 1 μm). (E) Representative images of HE staining and Nissl-staining in the ischemic penumbra 24 h after reperfusion. (F) Relative contents of SOD, CAT, GSH, MDA, and ROS in the right (ischemic) hemisphere of the sham-operation group and ischemia/reperfusion group before and after treatment. (G) Expression of Iba1, TNF-α, CD31, Ki67, ACE, ACE2, and TFEB in the cerebral ischemic penumbra analyzed by immunohistochemistry. (H) Quantitative statistics of the results of immunohistochemistry and immunostaining of angiogenesis (red: CD31; green: Ki67; blue: DAPI; scale bar = 100 μm) in the ischemic penumbra 24 h after reperfusion. (I) Representative paths during the Morris water maze spatial probe test. (J) Number of platform-crossings of rats in 60 s during the space exploration test. (K) Escape latency and swimming distance of rats varied with training times during the spatial navigation test. (L) Representative paths of different groups of rats in Y maze and open-field test. (M) Spontaneous alternation rate of rats in Y maze. (N) Standing times of rats in open field test. Data were expressed by mean ± SEM (*n* = 3). ^*#*^*P* < 0.05, ^*##*^*P* < 0.01, ^*###*^*P* < 0.001 compared with the control group; ^*^P < 0.05, ^**^P < 0.01, ^***^P < 0.001 compared with the model group.

After it had been proven experimentally that SHR could effectively deliver RT to the cerebral ischemic region, further studies were conducted to evaluate the therapeutic effect of this delivery system on I/R injury by a tMCAO model. First, the infarction area (by TTC stain) was visualized 24 h after reperfusion and analyzed by ImageJ software. As shown in Figure 4A–C, tail vein administration of 0.1 mg/kg RT and 0.1 mg/kg HA-RT almost had almost no influence on the cerebral infarct volume and the mNSS score compared with the model group. However, when 0.1 mg/kg HA-RT was administered intracerebroventricularly, the cerebral infarct volume and the mNSS score were significantly improved, indicating that BBB was the biggest obstacle for HR to exert its neuron-protective effect. In contrast, after connection with SS-31, 0.1 mg/kg SHR greatly decreased the volume of cerebral infarction compared with HR at same dosage (the mean infarct volume decreased from 17.8% to 4.2%), which was even similar to the protective effect of the same dose of intracerebroventricular injection group. Accordantly, similar results were obtained with the modified neurological deficit score in rats (Figure 4C). Notably, tail vein administration of 0.5 mg/kg SHR was more effective and even almost completely reversed the infarct injury. These results further demonstrate that the amount of HR delivered into the brain was significantly elevated after connection with SS-31, and SHR had a potent therapeutic effect on the neurological damage caused by ischemic stroke. Subsequently, electron microscopy was also applied to reveal the details of the drug against the cerebral ischemic injury. Nuclear atrophy, severe vacuolation, and mitochondrial swelling were observed in the penumbra of the tMCAO rat. As shown in Figure 4D, the nucleus of the SHR group was nearly normal: the intracellular arrangement was orderly, and the mitochondria were partially restored to normal and partially prolonged, which was a manifestation of hyperfunction. Further experiments revealed more information on the changes in histology and molecular biology. As shown in Figure 4E, after drug treatment, the number of the neurons impaired by chronic ischemia in the cerebral cortex and hippocampus area decreased, while the neurons in the model group were scattered with obvious loss of cells. It was also shown that, after treatment with SHR, the cell apoptosis decreased and Nissl bodies were recovered (Figure 4E). In addition, HE staining was performed on the paraffin sections of the heart, liver, spleen, lung, and kidney of the tMCAO rats in each group, and the results were almost indistinguishable, indicating that the micelles had high biological safety (Figure S2).

Considering that ischemia usually leads to malignant microenvironment, we also detected the oxidant, inflammatory, and angiogenesis situation in the penumbra of the tMCAO rat after SHR treatment. According to the results in Figure 4F, the ROS level increased significantly in the model group, while the enhancement was significantly inhibited by SHR treatment (*P* < 0.001). Accordingly, as the final product of peroxidation reaction between free radicals and lipids, the content of MDA was obviously reduced by SHR treatment (*P* < 0.01). Moreover, the contents of CAT, SOD, and GSH in the model group were significantly decreased compared with the sham group, while SHR could significantly restore the levels of these antioxidant (*P* < 0.001), indicating that SHR had a strong antioxidant effect. The immunohistochemical results in Figure 4G,H showed that the levels of the inflammatory factors Iba1 and TNF-α significantly increased after cerebral ischemia-reperfusion, and the angiogenesis factors CD31 and Ki67 increased compensatively. In contrast, after treatment with 0.1 mg/kg SHR, the expressions of Iba1 and TNF-α were significantly decreased, while the expressions of CD31 and Ki67 were further increased. It is worth mentioning that although the overall expressions of ACE and ACE2 in the model group were low, there was still a slight increase, and the increase of ACE was higher than that of ACE2. However, after administration, there was no significant change in the expression of ACE compared with the model group, but the expression of ACE2 was significantly increased. Since ACE2 has been reported to be the factors promoting anti-inflammatory and vascular normalization in ischemia, whereas ACE usually exerts the opposite effect,^31^ our results indicate that the anti-inflammatory and pro-angiogenic effects of SHR may be related to its effect on the expression of ACE2. In addition, the overexpression of TFEB during ischemia-reperfusion is often considered to be a manifestation of injury, but it is puzzling that SHR did not decrease the expression in the penumbra (Figure 4G).

Last but not least, we evaluated the behavioral functions of tMCAO rats with or without SHR treatment, since RT has been reported to improve cognitive dysfunction caused by various disease factors.^32,33^ The learning and memory functions of rats were detected by the Morris water maze, and the cognitive function was accessed by the Y-maze and open field test. As shown in Figure 4I,L, the total swimming distance and latency of the model group in the navigation experiment were significantly higher than those of the sham group, while the results of the SHR group was similar to those of the sham group (Figure 4K). In addition, in the space exploration experiment, the number of cross-platforms in the model group was much less than that in the sham group, while that in the SHR group was significantly improved (Figure 4J). Moreover, there was no significant difference in the total movement distance between the rats in the Y-maze and open field test, but the spontaneous alternation rates and upright numbers were significantly recovered after SHR treatment (Figure 4M,N). All these results suggest the great potential of SHR to recover ischemia-injured behavioral functions.

### SHR Promotes Vascular Normalization and Angiogenesis via the ACE2/Ang1-7 Signaling Pathway after Cerebral Ischemic Injury in Vitro and in Vivo

**Figure 5:**
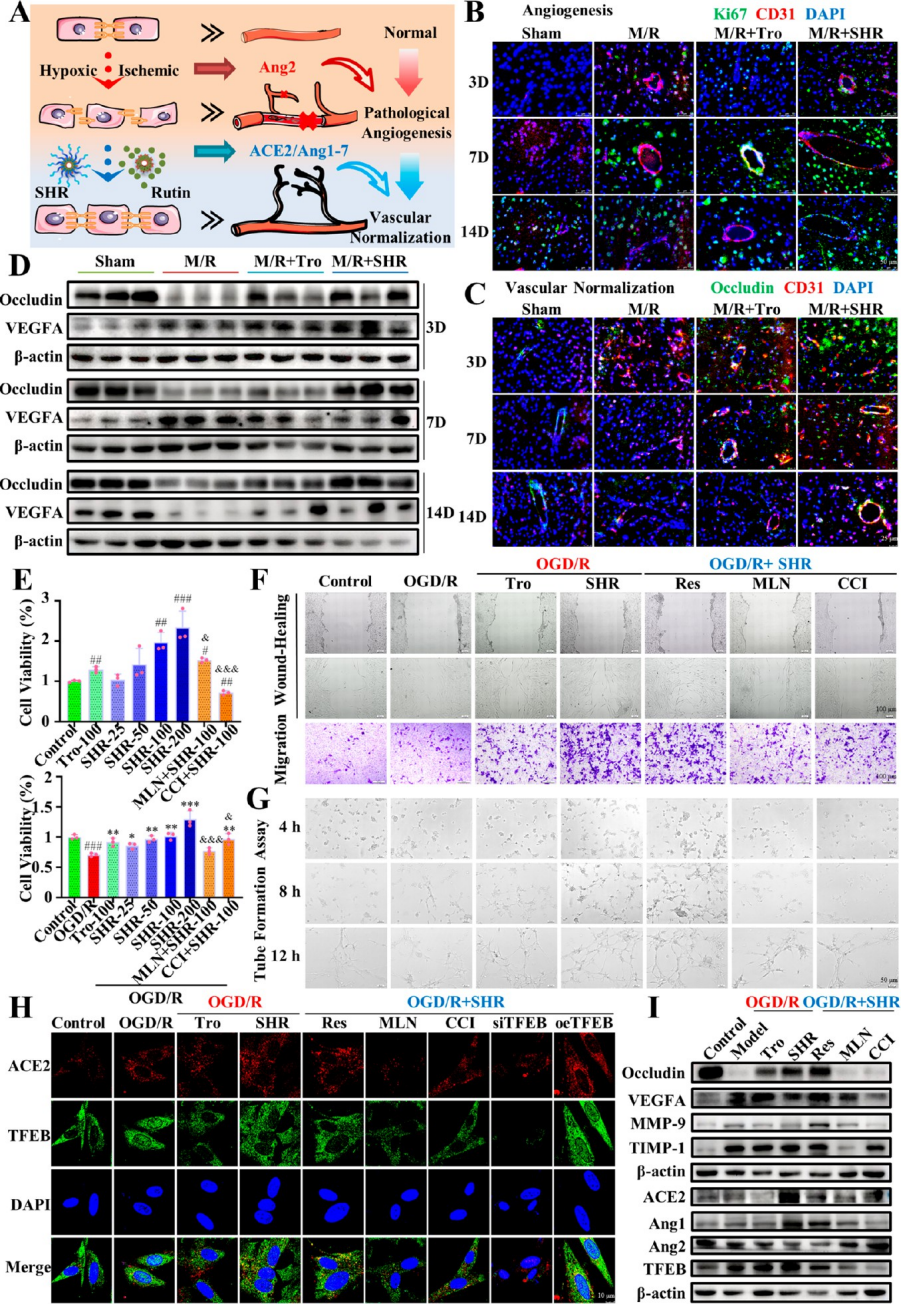
SHR micelles promoted the angiogenesis and vascular normalization in cerebral ischemic tissues and primary rBMECs. (A) Schematic diagram of the effect of SHR micelles on blood vessels. (B) Immunostaining of angiogenesis in the ischemic penumbra at 3, 7, 14 days after reperfusion (red: CD31; green: Ki67; blue: DAPI; scale bar = 50 μm). (C) Immunostaining of vascular normalization in the ischemic penumbra at 3, 7, 14 days after reperfusion (red: CD31; green: occludin; blue: DAPI; scale bar = 25 μm). (D) Levels of VEGFA and occludin in the cerebral ischemic penumbra at 3, 7, 14 days after reperfusion analyzed by Western blotting. (E) Promoting proliferation and anti-OGD damage of SHR micelles against rBMECs cells. (F) SHR micelles promoted the migration of rBMECs cells. (G) SHR micelles promoted the tube formation in vitro. (H) Expression and distribution of ACE2 and TFEB in rBMECs cells were observed by confocal immunofluorescence microscopy (red: ACE2; green: TFEB; blue: DAPI; scale bar = 10 μm). (I) Levels of occludin, VEGFA, MMP2, TIMP1, ACE2, Ang1-7, Ang2, and TFEB, analyzed by Western blotting. Data were expressed by mean ± SEM (*n* = 3). ^#^*P* < 0.05, ^##^*P* < 0.01, ^###^*P* < 0.001 compared with the control group; *P < 0.05, **P < 0.01, ***P < 0.001 compared with the model group; ^&^*P* < 0.05, ^&&^*P* < 0.01, ^&&&^*P* < 0.001 compared with the SHR group.

Previous reports have shown that endothelial cells might begin to proliferate as early as 12–24 h after ischemia and persist for up to several weeks.^34^ Clinical data also suggest that angiogenesis is active at 3 to 4 days after stroke, and the number of functional vessels may be correlated with the length of survival.^35^ However, angiogenesis induced by ischemia-reperfusion is compensatory and usually dysfunctional, leading to the risk of second stroke or bleeding.^36^ Therefore, we evaluated the potential of SHR on angiogenesis and vascular normalization by immunofluorescence staining to detect the colocation of CD31 and Ki67, colocation of CD31 and occludin on days (PSD) 3, 7, and 14 after stroke (Figure 5A). As shown in Figure 5B,C, compared with the sham group, the vascular endothelial cells in the infarcted area of tMCAO rats proliferated compensatively after MCAO injury, and tight junction proteins were significantly reduced. Notably, proliferative endothelial cells coexpressing CD31 and Ki67 appeared in the penumbra of the SHR-treated rats, accompanied by a significant increase in the level of the tight junction protein occludin. However, the Troxerutin-treated rats did not show such a dramatic improvement. In this study, Troxerutin, a RT derivative and a clinical blood-stimulating drug,^37^ was applied as the control drug. Next, to further verify the positive effect and potential mechanism of SHR on angiogenesis and normalization, proteins from the penumbra tissues were extracted on the PSD 3, 7, and 14 to determine the expression levels of VEGFA and occludin by Western blotting. As shown in Figure 5D and Figure S3A, the expression of VEGFA was abnormally increased on the third and seventh day after ischemia-reperfusion but significantly lower than that in the sham group on the 14th day. The expression of occludin was much lower than normal on the third, seventh, and 14th days, suggesting that angiogenesis induced by ischemia-reperfusion was compensatory and dysfunctional. However, it is inspiring that the SHR-treated rats not only maintained the high expression of VEGFA but also promoted the expression of occludin compared with the model group. Meanwhile, the effect of the same dose of Troxrutin was not good as that of the SHR group. These results confirm the proangiogenic and vascular normalizing effects of SHR micelles after stroke.

To further explore the mechanism of SHR micelles on angiogenesis and vascular normalization, we established an in vitro angiogenesis model. First, we detected the proliferative effect of different concentrations of SHR micelles on normal primary rat brain microvascular endothelial cells (rBMECs) and on oxygen-glucose deprivation-reperfusion (OGD/R). The pro-proliferative and repair effects of 25 μM, 50 μM, 100 μM, 200 μM SHR on rBMECs were concentration-dependent, while the effect of 100 μM Troxerutin was only equivalent to that of 50 μM SHR (Figure 5E). After 2 h of incubation with inhibitors of ACE2 or TFEB, the effect of SHR was significantly reduced, indicating that the protective effect of SHR on endothelial cells was closely related to ACE2 and TFEB. Further wound-healing assay and Transwell migration experiments (Figure 5F) showed that 100 μM SHR could greatly increase scratch healing and cell migration, which was much better than Troxrutin at the same concentration, suggesting the great proliferative effect on vascular endothelial cells. Pretreatment with Resorcinolnaphthalein (Res, the activator of ACE2) for 2 h slightly increased the effect of SHR, while pretreatment with MLN-4760 or CCI-779 (inhibitors for ACE2 and TFEB, respectively) for 2 h both significantly blocked the effect of SHR, indicating that ACE2 and TFEB exert positive roles in SHR-induced proliferation. Since the second step of angiogenesis after endothelial cell proliferation is tube formation, we further analyzed the tube formation ability on OGD rBMECs. As shown in Figure 5G, structurally unstable vessels were easily formed and dissociated in the model group within 12 h, while SHR treatment both greatly improved the number of tubes formed and the structural stability. In contrast, the effect of Troxrutin at the same concentration was so good. Furthermore, pretreatment with MLN-4760 or CCI-779 significantly blocked the effect of SHR, suggesting that ACE2 and TFEB play a key role in SHR-facilitated tube formation, which was also confirmed by Western blotting (Figure 5I and Figure S3B).

In order to further explore the mechanism of action of these two factors, we used laser confocal microscopy to observe the expression changes of ACE2 and TFEB in rBMECs by means of the knockdown and overexpression of genes as well as the activators and inhibitors of signaling. As shown in Figure 5H, after OGD/R, the bright fluorescent spots of ACE2 increased slightly, and the nuclear translocation of TFEB increased significantly. After treatment with 100 μM Troxerutin, the bright spots of ACE2 were further increased, but the nuclear translocation of TFEB was decreased compared with the model group. In contrast, after treatment with 100 μM SHR, the fluorescent spots of ACE2 were dramatically increased, while the nuclear translocation of TFEB was still significantly higher than that of the control group. These results suggest that ACE2 could be the major target of SHR, while TFEB was only slightly activated after drug treatment. Incubation with Res (activator of ACE2) could obviously increase the fluorescence of ACE2 but almost had no significant influence on the nuclear translocation of TFEB, whereas incubation with MLN-4760 (inhibitor of ACE2) significantly reduced both the fluorescence of ACE2 and the nuclear translocation of TFEB. CCI-779, an inhibitor of TFEB, also showed the similar effect at the same time, suggesting a positive interaction between SHR-activated ACE2 and TFEB. The above results were also confirmed by the knockdown (si-TFEB) and overexpression of TFEB. The concerning Western blotting results of rBMECs are shown in Figure 5I and Figure S3B. It can be seen that after OGD/R injury, the expression levels of VEGFA, TFEB, and Ang2 were increased significantly, while the expression levels of ACE2 and its downstream Ang1 were increased slightly, and the expression level of occludin decreased significantly. After treatment with Troxerutin, SHR or Res, the expressions of the above proteins increased. However, incubation with MLN-4760 or CCI-779 substantially reduces their expression levels. All these results are consistent with the fact that the promotion of angiogenesis and stabilization of vascular structure by SHR was comediated by TFEB and ACE2.

It has been reported that the expression and nuclear translocation of TFEB are significantly enhanced in the early stage of ischemia and gradually attenuated in the later stage.^38^ Little is known that TFEB not only mediates cell injury and repair as a key factor in the autophagy lysosome pathway but also positively regulates angiogenesis after stroke, such as promoting endothelial cell proliferation and migration.^39,40^ Our experimental results show that SHR can reduce the overexpression of TFEB at the early stage of ischemia, inhibit the reperfusion injury mediated by it, and enhance the expression of TFEB at the late stage of ischemia, thereby persistently exerting an effect on promoting angiogenesis. The expression of Ang2 is abnormally increased in the ischemic tissue, which mediates pathological angiogenesis and vascular leakage, and continuously releases vascular remodeling and instability factors downstream.^11,12,41^ In this study, after the RT released by the delivery system reaches the injury area of the brain, it can rapidly activate the ACE2 signaling pathway to degrade Ang2 into angiotensin 1-7 because of its high affinity with the ACE2 receptor on cell membrane. As a physiological antagonist of Ang2, Ang1-7 is able to inhibit the apoptosis of endothelial cells and stabilize the structure and function of new blood vessels, thereby promoting vascular normalization and improving the blood oxygen supply in the injury area. Given the results above, the effect of SHR on TFEB and ACE2 may be the core mechanism for promoting angiogenesis and vascular normalization.

### SHR Micelles Inhibit Cerebral Neuroinflammation by Regulation of Microglia Transformation via the ACE2/Ang1-7 Signaling Pathway

**Figure 6:**
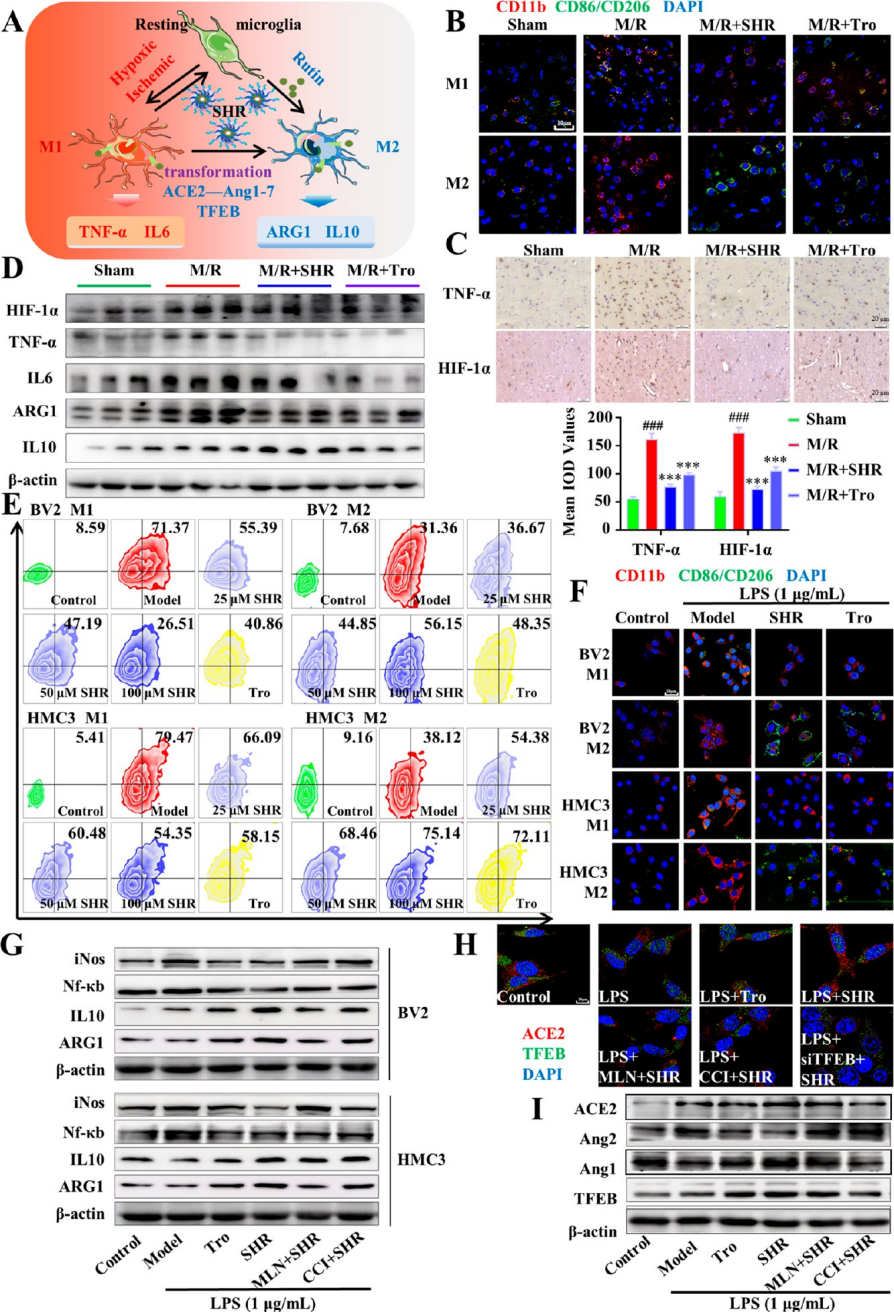
SHR micelles promoted microglial transformation and their anti-inflammation effect in vitro and in vivo. (A) Diagram of anti-inflammatory effect of SHR micelles. (B) Immunostaining of M1 and M2 microglia phenotype (red: CD11b; green: CD86/206; blue: DAPI; scale bar = 10 μm) in the ischemic penumbra 3 days after reperfusion. (C) Expressions of TNF-α and HIF-1α in the cerebral ischemic penumbra 3 days after reperfusion were analyzed by immunohistochemistry. (D) Levels of pro-inflammation and anti-inflammation related factors in the cerebral ischemic penumbra 3 days after reperfusion were analyzed by Western blotting. (E) Flow cytometry analysis of the M1 and M2 phenotypes of BV2 and HMC3 cells treated with SHR micelles and Troxerutin (Tro). (F) Expression of CD11b (red) and CD86/206 (green) was detected in microglia by confocal microscopy. (G) Changes in protein expression of iNOS, NF-κB, IL-10, ARG1 induced by SHR treatment. (H) Expression and distribution of ACE2 and TFEB in cells were observed by confocal microscopy with immunofluorescence. (I) Changes in protein expression of ACE2, Ang1, Ang2, and TFEB by different drug treatments. Data were expressed by mean ± SEM (*n* = 3). ^*#*^*P* < 0.05, ^*##*^*P* < 0.01, ^*###*^*P* < 0.001 compared with the control group; ^*^P < 0.05, ^**^P < 0.01, ^***^P < 0.001 compared with the model group.

Inflammation is the major mechanism of ischemic injury, in which the polarization of microglia plays an important role in the infarct cerebral area. In the pathological environment, a large number of microglia are activated into M1 phenotype which secrete pro-inflammatory factors such as TNF-α, IL-6, thus causing severe vascular inflammation.^42^ The transformation from M1 to M2 is considered as a target that suppresses neuroinflammation and promotes angiogenesis.^43^ It is well-known that Ang1-7, as a product of ACE2, can drive the transformation of microglia from M1 to M2 phenotype.^15,18^ Thereby, given the great effect of SHR on ACE2, we speculate that SHR should have a strong anti-inflammatory effect, as shown in the schematic diagram in Figure 6A. First, the phenotype of microglia in the ischemic penumbra 3 days after reperfusion was detected by immunofluorescence assay. As shown in Figure 6B, compared with the large amount of M1 type microglia in the model group, SHR significantly depressed the M1 type and increased M2 type. Next, microglia markers TNF-α and HIF-1α were analyzed by immunohistochemistry (Figure 6C). It can be seen that inflammatory cells with low intensity were observed in the cortex and hippocampus of the sham group, while they were densely expressed in the model group, especially in the cortex. SHR significantly decreased the expression of inflammatory cells, and the effect was better than that of Troxerutin. Western blotting analysis also revealed that SHR had a good anti-inflammatory effect by promoting the conversion of M1 to M2 type (Figures 6D and S4A). It is also obvious that SHR significantly inhibited the level of inflammatory factor IL-6 and increased the levels of anti-inflammatory factors ARG1 and IL-10 in the ischemic penumbra.

To further examine the anti-inflammatory mechanism of SHR, we constructed microglia activation models by inducing the polarization of mouse microglia BV2 and human microglia HMC3 with lipopolysaccharide (LPS), respectively. As shown in Figure 6E,F, after treatment with LPS for 24 h, the proportion of M1 phenotype cells (CD86^+^) to total activated state cells (CD11b^+^) was significantly increased, while the proportion of M2 phenotype cells (CD206^+^) to CD11b^+^ was increased in the presence of SHR or Troxerutin. Considering the extraordinary effect of SHR on ACE2/TFEB in vascular endothelial cells, we also determined the role of ACE2 and TFEB in the anti-inflammatory process of SHR. As shown in Figures 5G and S4B,C, the expressions of inflammatory factors iNOS and NF-κB were significantly inhibited after SHR treatment, while the expressions of anti-inflammatory IL10 and ARG1 were effectively enhanced in both BV2 and HMC3 cells. It is also shown that ACE2 inhibitor MLN-4760 and TFEB inhibitor CCI-779 significantly reversed the effect of anti-inflammatory of SHR, suggesting that ACE2 and TFEB were also involved in regulating the polarization and transformation of microglia. Next, the interaction between ACE2 and TFEB in microglia was further assessed. As shown in Figures 6H,I and S4D, SHR significantly promoted the nuclear translocation of TFEB and enhanced the fluorescence intensity of ACE2. After the expression of TFEB was decreased (by its inhibitor CCI-779 or its siRNA), the expression of ACE2 was also decreased accordingly, which is consistent with the fact that ACE2 inhibitor MLN-4760 significantly depressed the expression and nuclear translocation of TFEB. Moreover, since Ang2 is generally recognized as an activator of M1 microglia, Ang1-7 can drive the transformation of microglia from M1 to M2, and that ACE2 can convert Ang2 to Ang1-7, we further detected the protein levels of Ang2 and Ang1 after cotreatment with CCI-779 and MLN-4760. Results also revealed a similar trend after inhibitors treatment, suggesting that these two regulators indeed have an interactive effect on microglial transformation in the anti-inflammatory effect of SHR.

### SHR Exerts a Neuron-Protective Effect by the Crosstalk between BMECs and Microglial Cells via ACE2 and TFEB Signals

**Figure 7:**
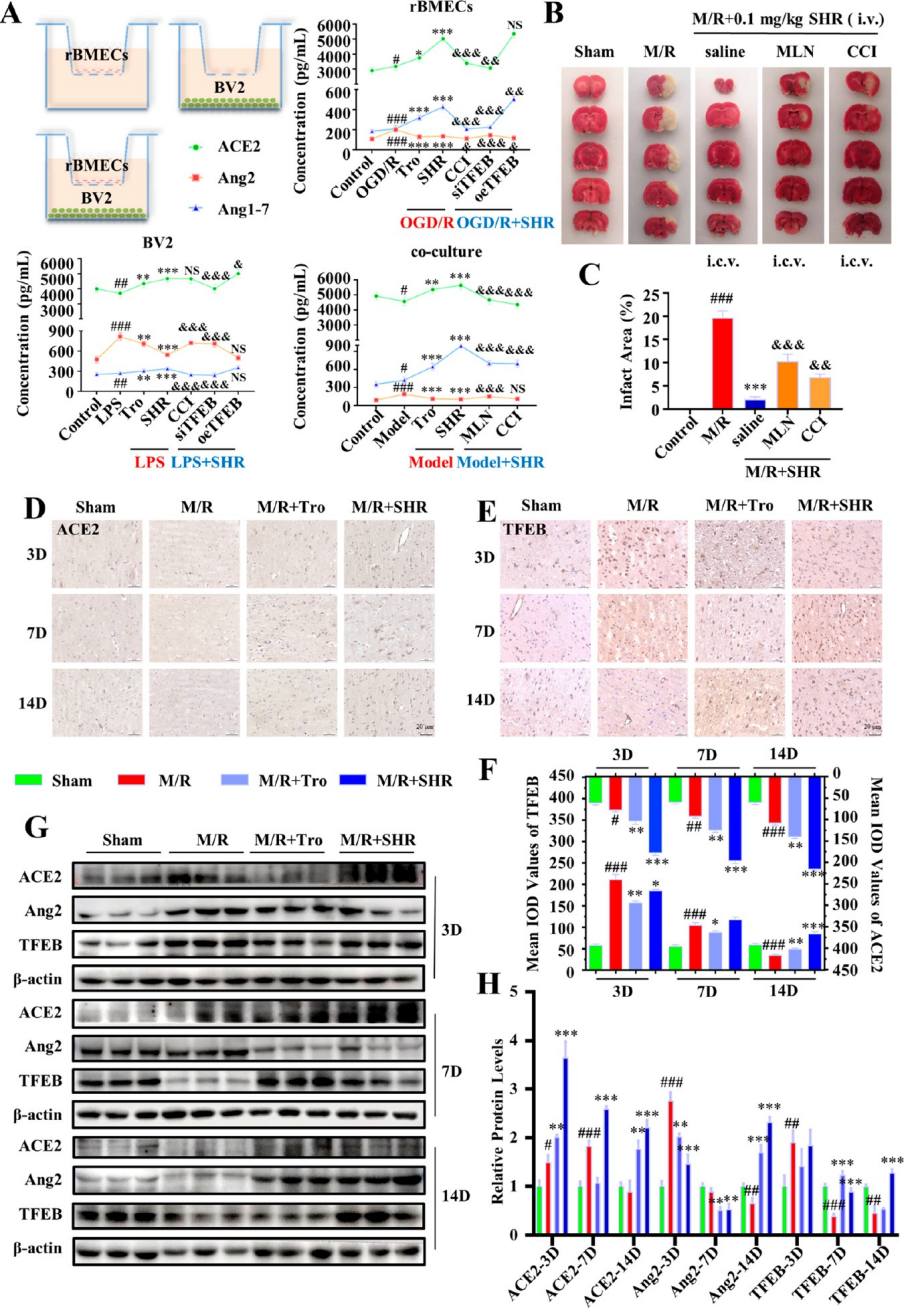
SHR induced crosstalk between BMECs and microglia via ACE2 and TFEB signaling. (A) Enzyme-linked immunosorbent assay (ELISA) was used to detect the contents of ACE2, Ang1-7, Ang2 in the supernatant of rBMECs or BV2 cells cultured alone and cocultured. (B) Representative TTC staining images of brain slices treated with SHR with or without the inhibitors of ACE2 and TFEB. (C) Quantification of the infarct area. (D) Expression of ACE2 in the cerebral ischemic penumbra 3, 7, 14 days after reperfusion was analyzed by immunohistochemistry (scale bar = 20 μm). (E) Expression of TFEB in the cerebral ischemic penumbra 3, 7, 14 days after reperfusion was analyzed by immunohistochemistry (scale bar = 20 μm). (F) Statistic analysis of the expression of ACE2 and TFEB. (G) Levels of ACE2, Ang2, and TFEB in the cerebral ischemic penumbra 3, 7, 14 days after reperfusion were analyzed by Western blotting. (H) Statistic analysis of the levels of ACE2, Ang2, and TFEB. Data were expressed by Mean ± SEM (*n* = 3). ^*#*^*P* < 0.05, ^*##*^*P* < 0.01, ^*###*^*P* < 0.001 compared with the control group; ^*^P < 0.05, ^**^P < 0.01, ^***^P < 0.001 compared with the model group; ^*&*^*P* < 0.05, ^*&&*^*P* < 0.01, ^*&&&*^*P* < 0.001 compared with the SHR group.

Our experimental results confirmed that SHR had angiogenic and vascular normalizing effects on BMECs and anti-inflammatory effects on microglia. More importantly, we discovered for the first time that ACE2 interacted with TFEB and that both factors played a key role in BMECs and microglial cells. Therefore, we further investigated whether the two major cells in the brain tissue play a role in the neuroprotection of SHR through the crosstalk of ACE2 and TFEB. First, we established three cell models using transwell chambers: single rBMECs (OGD model), single BV2 cells (LPS model), and coculture models of rBMECs and BV2 cells (OGD + LPS model) (Figure 7A). Enzyme-linked immunosorbent assay kit was employed to detect the contents of ACE2, Ang2, and Ang1-7 in the supernatant of the medium. It should be noted that the supernatant of the upper chamber was taken from the rBMECs group, and the supernatant of the lower chamber was taken from the BV2 and coculture groups. In each cell model group, the change trend of the contents of ACE2, Ang2, and Ang1-7 is similar to the previous experimental results; that is, SHR significantly enhanced the level of ACE2/Ang1-7 and decreased the level of Ang2. However, it is worth noting that compared with the BV2 group alone, the content of Ang2 decreased by SHR in the supernatant of the coculture group was further decreased, while the content of Ang1-7 was increased significantly. Likewise, the content of ACE2 and Ang1-7 increased by SHR in the rBMECs group was further elevated after coculture, which may be due to the crosstalk between the two kinds of cells. As a result, SHR caused rBMECs to secrete more ACE2 into the lower chamber, where ACE2 can substantially degrade Ang2 produced by LPS-activated BV2 cells, and the degradation product Ang1-7 can return to the upper chamber to provide more protection for rBMECs cells. Thus, the results of this coculture model demonstrate that SHR-activated ACE2 signaling plays an important role, especially under the crosstalk between BMECs and microglia.

In addition, the results of in vivo tMCAO experiments also demonstrate that microperfusion of MLN-4760 or CCI-779 in the right ventricle of rats could block the therapeutic effect of SHR and significantly increase the infarct volume (Figure 7B,C), further confirming that ACE2 and TFEB are important targets of SHR. Next, a time-course study of ACE2 and TFEB was further performed to reveal more details of these two targets. As shown in Figure 7D,F, the expression of ACE2 continued to increase with SHR treatment at 3, 7, 14 days after ischemia-reperfusion, while changes in the expression of TFEB seemed to be much more complicated. Especially after ischemia-reperfusion injury, the expression of TFEB first increased (day 3, day 7) and then gradually decreased until day 14, when it was lower than that of the sham group (Figure 7E,F). In contrast, after administration of SHR, the expression of TFEB started with a lower level than that in the model group but remained at a higher level on day 7 and 14. These results were verified by the Western blotting results shown in Figure 7G,H, suggesting that the interaction of the two factors might be more complex in the recovery process of ischemic stroke.

### SHR Exerts an Antitumor Effect by Vascular Normalization in Mice with Combination Model of Liver Cancer and Cerebral Ischemia

**Figure 8:**
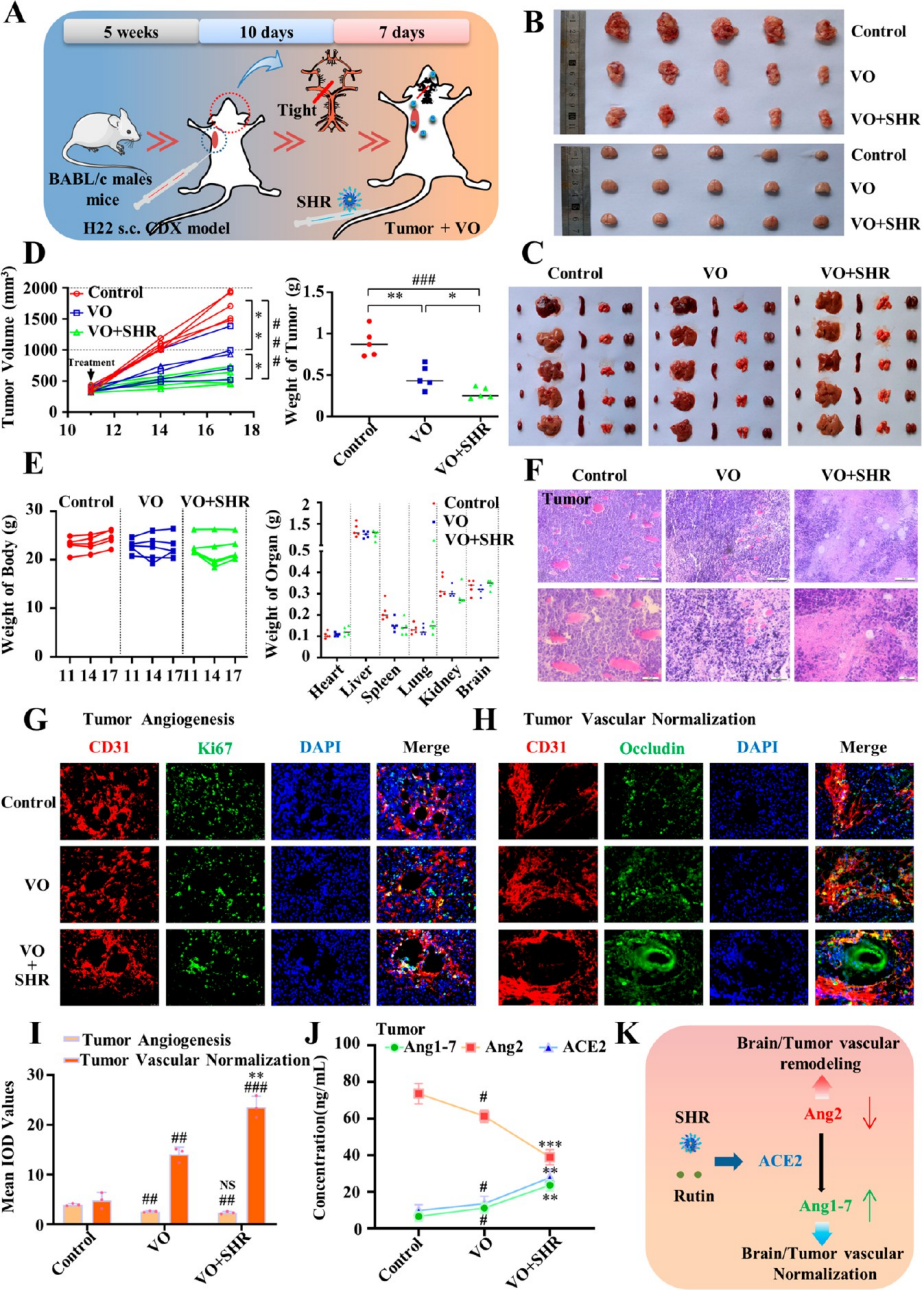
Antitumor effect of SHR micelles on vascular normalization in mouse model of cerebral vessel occlusion (VO) combined with liver cancer. (A) Construction and operation flowchart of chronic ischemia combined with H22 hepatoma in BABL/c mice. (B) Images of tumors and brains in each group. (C) Images of heart, liver, spleen, lung. and kidney of each group. (D) Growth curves and weight of tumors. (E) Body weight during treatment and the final weight of heart, liver, spleen, lung, kidney, and brain. (F) HE staining of tumor tissue. (G) Immunofluorescence staining of tumor angiogenesis (red: CD31; green: Ki67; blue: DAPI; scale bar = 25 μm). (H) Immunofluorescence staining of tumor vascular normalization (red: CD31; green: occludin; blue: DAPI; scale bar = 25 μm). (I) Statistic analysis of (G) and (H). (J) ELISA assay for ACE2, Ang1-7, Ang2 content in H22 hepatocellular carcinoma. (K) Schematic illustration of SHR micelles promoting the vascular normalization in liver cancer. Data were expressed by mean ± SEM (*n* = 5). ^*#*^*P* < 0.05, ^*##*^*P* < 0.01, ^*###*^*P* < 0.001 compared with the control group; ^*^P < 0.05, ^**^P < 0.01, ^***^P < 0.001 compared with the VO group.

Clinically, stroke patients usually suffer from concurrent diseases, so the selection of therapeutic agents needs to be more careful. Since SHR exhibited a great angiogenic effect on ischemic tissues, we are concerned that its application might lead to unnecessary tumor angiogenesis. Therefore, we constructed a model of chronic cerebral ischemia combined with liver cancer in BALB/c mice to evaluate the effect of SHR on tumors. The schematic diagram of the experimental process is shown in Figure 8A, the dissected images of tumors and main organs are shown in Figure 8B,C, and the calculated tumor volumes and weights are shown in Figure 8D. It can be seen that the tumor volume of the combined model was significantly smaller than that of the single liver cancer model but bigger than that of the SHR group. Meanwhile, the body weight and final organ weight of the mice in the SHR group (Figure 8E) were at normal levels, suggesting that SHR had a certain antitumor effect without any toxicity. To further analyze the safety of SHR, BALB/c mice were used to detect LD_50_ by the up-and-down method.^44^ The calculated LD_50_ was 56.25 mg/kg (Table S1), which was 100 times the effective dose, suggesting that SHR has great safety in future clinical applications.

The antitumor mechanism of SHR was further studied. According to the HE staining results in Figure 8F, there were more apoptotic cells in the combined model tumors with SHR treatment. As shown in Figure 8G,H, the immunofluorescence results showed no significant difference in angiogenesis (Ki67^+^/CD31^+^), significant difference in vascular integrity (Ki67^+^/Occludin^+^) among the three groups. According to the data in Figure 8I, when the level of tumor neovascularization was similar, the level of normalization was increased in the combined model group, and the effect was more significant in the SHR group, suggesting that vascular normalization might be the antitumor mechanism of SHR. Given the effect of ACE2 on vascular normalization, we further detected the expressions of ACE2, Ang1-7, and Ang2 in tumor tissue homogenates by an enzyme-linked immunosorbent assay, and the results (Figure 8J) showed that the content of Ang2 in tumor tissue was decreased by the combined ischemic model, and SHR treatment further depressed its expression. Meanwhile, the contents of ACE2 and Ang1-7 were significantly increased after SHR treatment, suggesting that the SHR-activated ACE2/Ang1-7 signaling also played a positive role in vascular normalization. It is known that there are many pathological blood vessels in tumor tissues, and the hypoxic environment is harsh, which leads to difficulties in treatment.^45−47^ Nowadays, antitumor research has started to focus on strategies to alleviate tumor hypoxia.^9,10,48^ Although the results in this study showed that SHR had no significant inhibitory effect on tumor angiogenesis, the enhanced expression of tight junction proteins implied that the pathological vessels tended to be normalized, which may facilitate further antitumor therapy. Our experimental results confirm that SHR is suitable not only for the treatment of cerebral ischemia but also for the treatment of sophisticated diseases (Figure 8J).

## Conclusions

In summary, a triple-targeted delivery system based on hyaluronic acid and rutin, SHR, was successfully developed for the treatment of cerebral ischemia in this study. With the help of a short peptide, SS31, and CD44-mediated endocytosis, this delivery vehicle can effectively penetrate the BBB, target the injured cerebral site, and then enrich the injured mitochondria. The hyaluronidase 1-mediated degradation and the acidic environment synergistically promoted the sustained release of rutin in the cerebral ischemic region. We discovered for the first time that rutin could effectively bind ACE2. Our experimental results suggest that SHR micelles exerted outstanding anti-inflammation, antioxidation, angiogenesis, and vascular normalization effects, which synergistically restored the damaged penumbra tissue by activating the signals of ACE2 and TFEB. It was also confirmed that ACE2 mediated the crosstalk of BMECs and microglia in the process of SHR treatment. Hence, the effect of SHR on vascular normalization shall provide the possibility for the treatment of combined diseases such as cerebral ischemia plus cancer.

## Methods

### Materials

All the chemicals and organic solvents used for synthesis in this study were obtained from Aladin Chemical Reagent Inc. (Shanghai, China) and used directly without purification or distillation unless otherwise specified. Hyaluronic acid (HA) (MW: 8k Da) was purchased from Freida Co., Ltd. 2,3,5-Triphenyltetrazolium (TTC) was purchased from Sigma (St Louis, MO, USA). MTT [3-(4,5-dimethylthiazol-2-yl)-2,5-diphenyltetrazolium bromide] was purchased from Sigma (St Louis, MO, USA). PINK1, P62, and LC3 were purchased from Affinity Biotechnology Co., Ltd. (Shanghai, China). Parkin, HIF-1α, TFEB, CD44, hyaluronidase-1, and p-mTOR were purchased from Bioss Biotechnology Co., Ltd. (Beijing, China). AKT and p-AKT were purchased from Cell Signaling Technology Co., Ltd. (Shanghai, China). mTOR was purchased from Wuhan Boster Bioengineering Co., Ltd. (Wuhan, China).

### Synthesis of HA-RT and SS31-HA-RT

HA (0.379 g, 1.0 mmol) was dissolved in 4 mL distilled water (ddH_2_O) to form a homogeneous solution, followed by the addition of 0.767 g EDC·HCl (4.0 mmol). After the solution was stirred at 0 °C for 2 h, a solution of 0.916 g RT (1.5 mmol) in 3 mL DMF was added dropwise. After the reaction was continued at room temperature for 12 h under argon, the reaction solution was transferred into a dialysis tubing (MWCO 3.5 kDa) to be dialyzed against ddH_2_O for 24 h, filtered through a 0.45 μm pore-sized microporous membrane, and lyophilized to obtain the desired product HA-RT (0.384 g, yield 90%). The molecular structure and RT grafting ratio (13%) were determined by ^1^H NMR (D_2_O, 300 MHz). HA-RT (0.108 g, 0.25 mmol) was dissolved in 1 mL ddH_2_O, followed by the addition of EDC·HCl (0.192 g, 4.0 mmol). The solution was stirred at 0 °C for 2 h, and then a solution of SS31 (0.016 g, 0.025 mmol) in 0.75 mL DMF was added dropwise. After the reaction proceeded under argon protection for 12 h, the reaction solution was transferred into a dialysis tubing (MWCO 3.5 kDa) to be dialyzed against H_2_O for 24 h, filtered through a 0.45 μm pore-sized microporous membrane, and freeze-dried to obtain the product SS31-HA-RT (0.104 g, yield 96%). The molecular structure and SS31 grafting ratio (5%) were determined by ^1^H NMR (D_2_O, 300 MHz).

### Cell Culture and Treatment

The brains from 4–10-day-old Sprague–Dawley rats were used to obtain brain microvascular endothelial cells (rBMECs). Briefly, brains were first placed in ice-cold PBS. After surface vessels and meninges were removed, and cortex gray matter was minced and incubated at 37 °C for 25 min in D-Hank’s solution containing 0.05% trypsin. Then, the samples were filled with 150 μm nylon mesh and centrifugated at 800*g* for 5 min, and the pellet was resuspended in PBS containing 20% bovine serum albumin (BSA) and centrifuged at 2000*g* at 37 °C for 5 min. Next, the pellet containing microvessels was resuspended and incubated at 37 °C for 30 min in PBS containing 0.1% collagenase II and then collected by centrifugation at 800*g* for 5 min. Finally, the pellet containing rBMECs was washed twice with PBS and cultured in DMEM/F12 (1:1) medium containing 20% fetal bovine serum at 37 °C in 5% CO_2_ humidified atmosphere.

Human neuroblastoma cell line SH-SY5Y, human microglia cell line HMC3, mouse microglia cell line BV2, and mouse hepatoma cell line H22 were maintained in DMEM (HyClone) containing 10% FBS (HyClone), and then incubated in a humidified atmosphere of 5% CO_2_ at 37 °C.

### Oxygen Glucose Deprivation/Reoxygenation Model

The medium was replaced with glucose-free Earle’s balanced salt solution (NaCl 143 mM, KCl 5.4 mM, CaCl_2_ 1.8 mM, MgSO_4_·7H_2_O 0.8 mM, NaH_2_PO_4_·2H_2_O 2.6 mM, NaHCO_3_ 26.2 mM, HEPES 20.1 mM, pH adjusted to 7.4). Then, the cells were placed in a gastight incubation chamber and flushed with gas (5% CO_2_/95% N_2_) for about 30 min, before the inlet and outlet valves of the chamber were closed. Next, the chamber was placed into a humidified incubator at 37 °C for 2 h. During OGD, the concentration of oxygen in the medium (<2.22 ± 0.10) ppm) was monitored with an oxygen electrode (East China University of Science and Technology, Shanghai, China). After 2 h, DMEN/F12 complete medium was added.

### LPS Induced Inflammation Model

LPS (Sigma) 1 μg/mL was incubated with BV2 and HMC3 cells for 24 h to generate the inflammatory model.

### Coculture of rBMECs and BV2 Cells

BV2 cells were seeded at a density of 45000 per filter (surface area, 0.33 cm; pore size, 0.4 μm; Corning Costar, Cambridge, MA, USA) on the bottom side of the transwell filter. The cells were then fed with DMEM containing 10% fetal bovine serum. After 24 h, rBMECs were seeded at a density of 30000 per filter on the upper, collagen-coated part of the filter. rBMECs + BV2 coculture was cultured to tight monolayers in DMEM. Then, the TEER was measured to evaluate the integrity of rBMECs monolayer. TEER (Ω/cm^2^) was determined using an electrical resistance system with a current-passing and voltage-measuring electrode (Millicell-ERS, Millipore, Bedford, MA, USA).

### Animals

Adult male Sprague–Dawley rats (220 to 250 g, 7 to 8 weeks) and male BALB/c mice (20 to 25 g, 6 to 7 weeks) were from the laboratory animal center of Henan Province (Zhengzhou, China). The experiments were carried out following the Principles of Laboratory Animal Care of Henan University (Kaifeng, China) and approved by the Animal Ethics Committee of Henan University (HUSOM2021-046).

### tMCAO Procedure

Focal cerebral ischemia was performed by transit middle cerebral artery occlusion (tMCAO). Rats were anesthetized using 1.5% isoflurane in 70/30 nitrogen/oxygen gas. After the right common, external, and internal carotid arteries were exposed, the origin of the middle cerebral artery (MCA) was blocked by a monofilament nylon suture (diameter about 0.26 mm) which was introduced into the external carotid artery, then, advanced along the internal carotid artery (ICA), approximately 18–20 mm from the carotid bifurcation, until occluding the origin of the MCA in the circle of Willis. Two hours after the MCAO, the suture was removed. During the experiment, the body temperature was maintained at 37 °C and the local cerebral blood flow (LCBF) of the middle cerebral artery territory was monitored by laser-Doppler fluxmetry (LDF) (MP150 starter system, BIOPAC system, Inc., USA). Sufficient occlusion of the MCA was determined by LDF (decrease of LCBF below 30% of baseline).

### Drug Administration

In the short term experiment, the animals were divided into ten groups (*n* = 3–6): Sham, model (MCAO with reperfusion, M/R), RT (0.1 mg/kg, i.v.) + M/R, HA-RT (HR, 0.1 mg/kg, i.v.) + M/R, HA-RT (HR, 0.1 mg/kg, i,c,v,) + M/R, SS-31-HA-RT (SHR, 0.025 mg/kg, i.v.) + M/R, SS-31-HA-RT (SHR, 0.1 mg/kg, i.v.) + M/R, SS-31-HA-RT (SHR, 0.5 mg/kg, i.v.) + M/R, MLN-4760 (MLN, 5 μL, i.c.v.)+ M/R + SS-31-HA-RT (SHR, 0.1 mg/kg, i.v.), and CCI-779 (CCI, 5 μL, i.c.v.) + M/R + SS-31-HA-RT (SHR, 0.1 mg/kg, i.v.). The mice were administered intraperitoneally at a dose of 0.1 mL/kg. In the sham operation group, the vessels were separated without thread plug and the same amount of normal saline was given. In the long term experiment, the animals were divided into three groups (*n* = 3–6). One hour after the MCAO, SS-31-HA-RT (SHR, 0.1 mg/kg, i.v.) were administered intraperitoneally at a dose of 0.1 mL/kg every 3 days. In the sham operation group, the vessels were separated without thread plug and the same amount of normal saline was given.

### Small Animal Imaging Technology

The animals were divided into three groups (*n* = 3–6). One hour after the MCAO, IR780-HA-RT (IHR, 0.1 mg/kg, i.v.) and IR780-SS-31-HA-RT (ISHR, 0.1 mg/kg, i.v.) were administered intraperitoneally at a dose t of 0.1 mL/kg to the rats. In the sham operation group, the vessels were separated without thread plug and the same amount of IR780-SS-31-HA-RT was given (ISHR, 0.1 mg/kg, i.v.). After 24 h, the fluorescence maps of drug accumulation and distribution in the brain of the three groups of rats were detected by a small animal imaging system (IVIS Lumina XRMS Series III, PerkinElmer).

### Measurement of Cerebral Infarct Volume, Neurological Scores

The cerebral infarct volume was measured by TTC (2, 3, 5-triphenyl-2*H*-tetrazolium chloride) staining. After the brain was removed, the slices (2 mm-thick) from the frontal pole to the occipital pole were obtained and stained using 2% TTC at 37 °C for 30 min. The infarct size of the brain was determined using an ImageJ (ver 1.37c, NIH, Bethesda, MD, USA). The infarct volume was calculated following the equation: Percentage of infarct area (%) = {[total infarct volume – (right hemisphere volume–left hemisphere volume)]/total brain volume} × 100%.

Twenty-four hours after tMCAO, a modified neurologic severity score (mNSS) was used to determine the neurological deficits of each animal by two observers who were blinded to the treatment. The rats were placed in a rotating cylinder, and the time of the animals on it was recorded. The experiment ended when the animal fell from the rungs or gripped the device and spun for two consecutive revolutions.

### Behavioral Evaluation by Morris Water Maze, Y Maze, and Open Field Test

On the 21st day, rats were tested with Y maze (ZS-MGY, Zhongshidichuang Technology Development Co., Ltd.). The Y-maze test relies on the innate tendency of rats to explore a novel environment. The apparatus consists of three arms, which were labeled as A, B, and C. Briefly, at 1 h post the last drug administration, the animals were subjected to the Y-maze task. The mice were placed in the center of the Y-maze facing the south arm B and were allowed to explore the maze freely for a period of 8 min. The number and the sequence of arm entries were recorded by an observed unaware of the treatment groups. Alternation behavior was defined as consecutive entries into all three arms, (i.e., ABC, CAB, or BCA but not BAB). The percentage of spontaneous alternation was measured as an index of working memory = [(number of alternations)/(total number of arm-entries-2)] × 100. The total number of arm entries was recorded as an index of locomotor activity

On the 22nd day, the open field was used to evaluate anxiety-like behaviors. The inner wall of the open-field reaction tank is painted black, and a digital camera is installed on the top, whose field of vision can cover the entire interior of the open-field. The spontaneous activity of animals was measured by open field experiment. The total distance of movement and upright times of rats were recorded within 5 min. The inner wall and bottom of the box were cleaned to prevent the residual information on the last animal (such as animal size, urine, smell) from affecting the next test result.

On the 23rd day, Morris water maze (MWM, ZS-001, Zhongshidichuang Technology Development Co., Ltd.) was used to test the learning and memory ability of the rats for 5 consecutive days. The experiment was divided into two parts: navigation experiment (acquisition period) and space exploration experiment (exploration period). The navigation experiment lasted for 4 days, and each rat was trained 4 times a day. The time to find the platform was recorded as the escape latency, and the average escape latency was measured 4 times a day for statistical analysis. The space exploration experiment lasted for 1 day. The platform was removed, and the number of times each animal crossed the platform location within 60 s, the time proportion and swimming distance proportion of the platform quadrant (target quadrant) were recorded as indicators to measure their learning and memory ability.

Biochemical Assays: After the short-term behavioral test, the mice were decapitated, and then the skulls were cut open to expose the brain from the dorsal side. The whole brain was quickly removed and homogenized in a 0.03 M sodium phosphate buffer pH 7.4 employing a homogenizer. The homogenate was used for the determination of reactive oxygen species (ROS), malondialdehyde (MDA), glutathione (GSH), and superoxide dismutase (SOD) by kits from Beyontime Institute of Biotechnology (Shanghai, China).

### Histopathological Study

Under deep anesthesia, the brains were perfused through a transcardial perfusion of 200 mL normal saline followed by 200 mL of 4% paraformaldehyde (PFA) in 0.1 M phosphate buffer (prefixation). Then, the brains were excised and sliced coronally into 3–5 mm-thick sections including the dorsal hippocampus, which were postfixed in 10% formalin at 4 °C for 72 h. Next, the samples were embedded in paraffin and 8 μm coronal sections (one from each five sections) were prepared using a rotary microtome (Leica Biosystems, Milan, Italy) and observed using light microscopy. The tissue sections stained with Hematoxylin and eosin (Solarbio, Beijing, China) and Nissl methods (0.5% cresyl violet). In this way, the sections were dehydrated through graded alcohols (70, 80, 90, and 100% × 2), mounted in neutral resins, and then analyzed using a light field microscope (Leica TCS SP8, Germany).

### Transmission Electron Microscope

The brain tissues of the penumbra in the sham operation group, the model group and the SHR administration group were cut into blocks and soaked in the electron microscope fixing solution for examination for mitochondria.

### Zebrafish Maintenance and Embryo Collection

In this study, wild-type AB zebrafish, and Tg (cmcl2: EGFP) and Tg (flk1: EGFP) transgenic zebrafish strains were used. Male and female zebrafish were kept separately under standardized conditions of 14 h illumination/10 h dark at 28 °C, and regularly fed with paramecium and brine shrimp. Healthy, mature zebrafish were placed in a mating tank at a male to female ratio of 1:1. The next day, male and female zebrafish mated to lay eggs, and fertilized eggs were obtained 2 h later. The fertilized eggs were collected, transferred to fresh zebrafish embryo culture solution, and incubated in a thermostatic controlled-light incubator at 28 °C.

### Neuroprotective Effect of SS-31-HA-RT on Zebrafish

At 120 hpf, 18 juvenile zebrafish of AB line were taken from each concentration group transferred into a 48-well plate with 1 juvenile zebrafish placed in each well. The concentration groups were set as follows: blank control group (zebrafish embryo culture water), modeling group (15 mM PTZ), and SS-31-HA-RT group (15 mM PTZ + 5, 10, 20 μM SS-31-HA-RT). Zebralab (Viewpoint, Lyon, France) was used to analyze the behavioral changes of zebrafish in each concentration group, and the average movement speed (mm/min) of juvenile zebrafish in 20 min was recorded.

### Antioxidant Effect of SS-31-HA-RT on Juvenile Zebrafish

At 24 h of development, the egg membrane was removed with 1.0 mg/mL chain enzyme protease E solution. Normal zebrafish embryos were selected under a stereomicroscope and transferred into 24-well culture plates with 10 embryos in each well and 3 replicates in each group. The concentration group was divided into blank control group (zebrafish embryo culture water), modeling group (5 mM MTZ), positive drug group (5 mM MTZ + 100 μL/mL vitamin C), concentration group (5 mM MTZ + 5, 10, 20 μM SS-31-HA-RT). Then it was cultured in 28 °C light culture. At 48 hpf, the fluorescent spots on zebrafish skin were observed by fluorescence photography under a stereomicroscope, and the number of fluorescent spots was counted by Image-Pro Plus software.

### Anti-Inflammatory Effects of SS31-HA-RT on Juvenile Zebrafish

Normal 72 hpf Tg (ZLYz-EGFP) transgenic zebrafish were selected under a stereological microscope and carefully transferred into 6-well plates with 30 juvenile fish in each well. The blank control group (zebrafish embryo culture water), model group (20 μM CuSO_4_), positive drug group (20 μM CuSO_4_ + 20 μM Ibuprofen) and sample group (20 μM CuSO_4_ + 5, 10, 20 μM SS-31-HA-RT) were set up, and each concentration group had 3 repeating wells. After the zebrafish were treated with different concentrations for 6 h, 20 μM CuSO_4_ was added. After treating zebrafish for 1 h, zebrafish embryo culture water was used to wash it for 3 times. Then, each zebrafish juvenile was photographed under a fluorescence microscope, and the number of macrophages was counted by Image-Pro Plus software.

### Effects of SS-31-HA-RT on Angiogenesis in Zebrafish

At 24 h of development, the egg membrane was removed with 1.0 mg/mL chain enzyme protease E solution. Normal zebrafish embryos were selected under stereomicroscope and transferred into 24-well culture plates with 10 embryos in each well and 3 replicates in each group. Concentration groups were set as Blank control group (zebrafish embryo culture water), modeling group (0.2 μg/mL PKT787), positive drug group (0.2 μg/mL PKT787 + 10 μL/mL DH), SS-31-HA-RT group (0.2 μg/mL PKT787 + 5, 10, 20 μM SS-31-HA-RT). Then it was cultured in 28 °C light culture. At 48 hpf, generation of intersegmental vessels (vessels ISVs) of zebrafish was observed by fluorescence photography under stereographic microscope, and the length of intersegmental vessels was calculated.

### TFEB Overexpression and si-TFEB Transfection

Cells were transfected with TFEB-overexpression and TFEB-siRNA plasmid by using Transfection Reagent (Kemix) according to the manufacture’s protocol. Forty-eight hours after transfection, the cells were collected for each experiment.

### Enzyme-Linked Immunosorbent Assay (ELISA)

The contents of ACE2, Ang1-7, Ang2 in the medium of rBMECs and BV2 cells were determined by ELISA kit according to the instruction. Briefly, the ACE2/Ang1-7/Ang2 working solution was first diluted to 6 standard-samples at concentrations of 0, 2, 4, 6, 8, 10 ng/mL to establish the standard curve. Next, the standard samples and test samples were added into a 96-well plate and precoated with antibody. Then, the plate was incubated at room temperature for 2 h and washed with washing buffer 5 times, followed by the addition of Avidin-HRP for 1 h of incubation and then washed 5 times. Finally, TMB substrate was added, followed by incubation for 15 min. After the stop solution was added, the absorbance at 450 nm was recorded with a microplate reader. The contents of ACE2, Ang1-7, Ang2 in the medium were calculated according to the standard curve.

### Cell Viability Assay

Cell viability was evaluated using MTT assay (Solarbio, Beijing, China). Briefly, rBMECs and SH-SY5Y cells were seeded into 96-well plates. Then, the cells were treated with OGD/R + 100 μM Troxerutin, OGD/R + 0, 25, 50, 100, 200 μM SHR, MLN-4760 + OGD/R + 100 μM SHR and CCI-779 + OGD/R + 100 μM SHR for 24 h, respectively. Next, 10 μL of MTT (5 mg/mL) solution was added to each well followed by incubation at 37 °C for another 4 h. The absorbance was measured at 570 nm using a Multiskan spectrum microplate reader (Thermo Scientific, Shanghai, China).

### Wound-Healing Assay

rBMECs were seeded in a six-well plate at a density of 1 × 10^6^ cells/well. After attachment overnight, the monolayers were wounded by scraping with a P20 micropipette tip and the cells were washed twice with PBS (pH 7.4). The medium was replaced with serum-free medium following the previous treatment. At the indicated times (0, 24 h) after scraping, the representative images were obtained by microscopy (Ningbo Sunny Instruments Co. Ltd. China).

### Cell Migration Assay

1× 10^5^ cells were seeded into the upper chamber in serum-free medium coated with Matrigel (BD Biosciences, San Jose, CA, USA). Then 600 μL corresponding medium containing 10% FBS was added into the lower chamber. After the same treatment as above, the cells remaining on the upper side were scrubbed with a cotton tip swab, while the cells on the bottom surface of the membrane were fixed with 4% paraformaldehyde and stained with 0.1% crystal violet. The number of cells in randomly selected fields was counted under a light microscope at 200× magnification.

### Tube Formation Assay

A 96-well plate was first coated with 30 μL of growth factor-reduced Matrigel (BD Pharmingen) and then incubated for 1 h at 37 °C. After the same treatment as above, rBMECs (1.5 × 10^5^ cells/mL) in DMEM/F12 were seeded into 96-well plates. After the plate was incubated for 12 h at 37 °C, the morphology of the cells was photographed and analyzed using a phase contrast inverted microscope. The total tube length was calculated using Image-Pro Plus software (Media Cybernetics).

### Up and down Method for LD_50_

The dose group was divided into 20, 30, 50, 55, and 60 mg/kg according to the primary study. Then, the experiment was conducted one by one from the dose group 20 mg/kg. If the mice died in 24 h after SHR treatment, the next animal was given a lower dose. And if the mice survived, the next animal was given a higher dose. Toxicity and death were recorded, and LD_50_ was calculated by AOT425 StatPgm program.

### Tumor Xenograft Combined with Chronic Cerebral Ischemia Model

Male BALB/c mice (5 weeks of age) were fed a standard diet and housed in a vivarium with controlled room temperature and humidity. A total of 4 × 10^5^ H22 cells were injected subcutaneously into the flank of mice. When the tumor size reached approximate 100 mm^3^, the mice were randomly divided into three groups (*n* = 5–7): model group (saline, equal volume of drug administration), common carotid occlusion (vessel occlusion, VO) group, and VO + SHR group. Body weight and tumor volume were measured every 2 days. Tumor volume was calculated according to the formula (length × width^2^)/2. After 7 days, the mice were euthanized and the final organ weight was determined.

Chronic cerebral ischemia injury model was established by permanent ligation of unilateral common carotid artery. Before the operation, all the animals were fasted for 8 h, anesthetized with 4% isoflurane in the air, and maintained sedated with 1–1.5% isoflurane during the operation. The rectal temperature was maintained at 37.0 ± 0.5 °C and heated with a heating pad. The common carotid artery was exposed by midline incision and ligated with 4–0 silk thread for one time. In the sham operation group, the rats received the same sham operation without ligation of the common carotid artery. The VO animals were randomly divided into three experimental groups: model group, VO group, and VO + SHR (0.2 mg/kg, i.v.) group. Finally, 5 rats in each group were utilized.

### Flow Cytometry

BV2 and HMC3 cells grew exponentially with a density of 4 × 10^5^ cells/plate. After overnight adherence, the cells were synchronized after 12 h. Then, the cells were divided into control group (only serum-free DMEM medium was added), model group (incubated with 1 μg/mL LPS for 24 h), treatment group (incubated with 1 μg/mL LPS for 24 h, followed by the addition of 25 μM, 50 μM and 100 μM SHR or 100 μM Troxerutin for another 24 h of incubation). After treatment, the cells were removed from the dish, resuspended, and fixed with 4% paraformaldehyde at room temperature for 30 min. After being washed with PBS, the cells were incubated with 5% BSA on ice for 2 h, and washed with PBS once again, followed by incubation with CD11b antibody and FITC-conjugated secondary antibody. After being washed, the cells were resuspended in cell-staining buffer and analyzed by flow cytometry (BD FACSVerse). The fractal expression of M1 and M2 was analyzed by dressing with CD11b antibody and CD86/CD206 and AF555/FITC-conjugated secondary antibody. Then, the cells were washed with PBS and analyzed by flow cytometry (BD FACSVerse). With the same method, the expression of CD44 and hyaluronidase-1 in SH-SY5Y cells before and after OGD was detected.

### Immunofluorescence

Cells were plated on glass coverslips and then fixed in 4% paraformaldehyde in PBS for 20 min after drug treatment. Then, the cells were washed twice with cold PBS and incubated with PBS containing 0.25% Triton X-100 for 10 min. After being blocked with 5% BSA for 30 min, the cells were incubated with primary antibodies in 0.5% BSA in a humidified chamber overnight at 4 °C. Subsequently, the cells were washed with TBST three times and incubated with goat anti-rabbit IgG (H+L)/FITC conjugated secondary antibody and goat anti-mouse IgG H&L/Alexa Fluor 555 conjugated secondary antibody at room temperature for 1 h, followed by incubation with 10 μg/mL DAPI for another 30 min. Signals were visualized and recorded using a Confocal Microscopy (Leica TCS SP8).

### Immunohistochemistry (IHC)

After drying at 60 °C in an incubator for 2 h, the 8-μm-thick paraffin embedded slices were dewaxed with xylene for 20 min twice. Then, the slices were soaked in 100%, 95%, 90%, 80%, and 70% ethyl alcohol for 5 min in sequence. After the slices were washed with PBS for 5 min three times, a citrate buffer solution (0.01 M pH 6.0) was used for antigen retrieval. Thereafter, the slices were cooled at room temperature for 20 min and then washed with PBS thrice for 5 min. After being permeabilization with 0.5% Triton X-100 for 30 min and three washing steps, the samples were blocked in 10% BSA for 1 h and incubated overnight at 4 °C with primary antibodies. On the next day, the 8-μm-thick paraffin embedded slices were reacted with avidin–biotin-peroxidase complex and DAB (Boster Bioengineering, Wuhan, China). Finally, the levels of target protein in the ischemic cerebral cortex were investigated using a light microscope at a magnification of 200×.

### Western Blot Analysis

The cells and brain tissues were collected and lysed with RIPA buffer (Solarbio, Beijing, China), with added 1 mM PMSF and 1% cocktail of protease inhibitors (Solarbio, Beijing, China). Protein concentrations were measured using the BCA reagent (Beyotime, Shanghai, China). For Western blotting analysis, an equal amount of protein was loaded for SDS-polyacrylamide gel electrophoresis. Then, the proteins were transferred to polyvinylidene difluoride (PVDF) membranes, which were then blocked with 5% skim milk for 2 h at room temperature. Next, the membrane was incubated with the indicated primary antibodies overnight at 4 °C. The membranes were then incubated with HRP lgG (H + L) secondary antibodies for 2 h at room temperature. Protein expression was visualized with an enhanced chemiluminescence reagent (BeyoECL Star, Shanghai, China).

### Statistical Analysis

All the experiments were performed at least in triplicates. All the data were presented as (means ± S.D.). Significant differences between the groups were determined by One-way ANOVA followed by Dunnett’s multiple comparison tests. *P*-values less than 0.05 were considered as statistically significant.

### Safety Statement

No unexpected or abnormally high safety hazards were encountered in the experiment.

## Supporting Information Available

Additional figures, including HE staining of heart, liver, spleen, lung, and kidney in rats 21 days after cerebral ischemia reperfusion, quantitative analysis of protein expression in Figure 5, and calculation of LD_50_ for SHR micelles (PDF)

## References

[ref1] Campbell B. C. V.; De Silva D. A.; Macleod M. R.; Coutts S. B.; Schwamm L. H.; Davis S. M.; Donnan G. A. Ischaemic stroke. Nat. Rev. Dis Primers 2019, 5 (1), 70 10.1038/s41572-019-0118-8. 31601801

[ref2] Emberson J.; Lees K. R.; Lyden P.; Blackwell L.; Albers G.; Bluhmki E.; Brott T.; Cohen G.; Davis S.; Donnan G.; et al. Effect of treatment delay, age, and stroke severity on the effects of intravenous thrombolysis with alteplase for acute ischaemic stroke: a meta-analysis of individual patient data from randomised trials. Lancet 2014, 384 (9958), 1929–1935. 10.1016/S0140-6736(14)60584-5. 25106063 PMC4441266

[ref3] Zhu Z.; Fu Y.; Tian D.; Sun N.; Han W.; Chang G.; Dong Y.; Xu X.; Liu Q.; Huang D.; et al. Combination of the Immune Modulator Fingolimod With Alteplase in Acute Ischemic Stroke: A Pilot Trial. Circulation 2015, 132 (12), 1104–1112. 10.1161/CIRCULATIONAHA.115.016371. 26202811 PMC4580515

[ref4] Sun Y.; Jin K.; Xie L.; Childs J.; Mao X. O.; Logvinova A.; Greenberg D. A. VEGF-induced neuroprotection, neurogenesis, and angiogenesis after focal cerebral ischemia. J. Clin. Invest. 2003, 111 (12), 1843–1851. 10.1172/JCI200317977. 12813020 PMC161428

[ref5] Hoffmann C. J.; Harms U.; Rex A.; Szulzewsky F.; Wolf S. A.; Grittner U.; Lattig-Tunnemann G.; Sendtner M.; Kettenmann H.; Dirnagl U.; et al. Vascular signal transducer and activator of transcription-3 promotes angiogenesis and neuroplasticity long-term after stroke. Circulation 2015, 131 (20), 1772–1782. 10.1161/CIRCULATIONAHA.114.013003. 25794850

[ref6] Kim I. D.; Cave J. W.; Cho S. Aflibercept, a VEGF (Vascular Endothelial Growth Factor)-Trap, Reduces Vascular Permeability and Stroke-Induced Brain Swelling in Obese Mice. Stroke 2021, 52 (8), 2637–2648. 10.1161/STROKEAHA.121.034362. 34192895 PMC8312568

[ref7] Pradillo J. M.; Hernández-Jiménez M.; Fernández-Valle M. E.; Medina V.; Ortuño J. E.; Allan S. M.; Proctor S. D.; Garcia-Segura J. M.; Ledesma-Carbayo M. J.; Santos A.; et al. Influence of metabolic syndrome on post-stroke outcome, angiogenesis and vascular function in old rats determined by dynamic contrast enhanced MRI. Journal of Cerebral Blood Flow & Metabolism 2021, 41 (7), 1692–1706. 10.1177/0271678X20976412. 34152893 PMC8221771

[ref8] Yang Y.; Torbey M. T. Angiogenesis and Blood-Brain Barrier Permeability in Vascular Remodeling after Stroke. Curr. Neuropharmacol 2020, 18 (12), 1250–1265. 10.2174/1570159X18666200720173316. 32691713 PMC7770645

[ref9] Park J. S.; Kim I. K.; Han S.; Park I.; Kim C.; Bae J.; Oh S. J.; Lee S.; Kim J. H.; Woo D. C.; et al. Normalization of Tumor Vessels by Tie2 Activation and Ang2 Inhibition Enhances Drug Delivery and Produces a Favorable Tumor Microenvironment. Cancer Cell 2016, 30 (6), 953–967. 10.1016/j.ccell.2016.10.018. 27960088

[ref10] Martin J. D.; Seano G.; Jain R. K. Normalizing Function of Tumor Vessels: Progress, Opportunities, and Challenges. Annu. Rev. Physiol. 2019, 81, 505–534. 10.1146/annurev-physiol-020518-114700. 30742782 PMC6571025

[ref11] Gurnik S.; Devraj K.; Macas J.; Yamaji M.; Starke J.; Scholz A.; Sommer K.; Di Tacchio M.; Vutukuri R.; Beck H.; et al. Angiopoietin-2-induced blood-brain barrier compromise and increased stroke size are rescued by VE-PTP-dependent restoration of Tie2 signaling. Acta Neuropathol 2016, 131 (5), 753–773. 10.1007/s00401-016-1551-3. 26932603 PMC4835530

[ref12] Mirando A. C.; Shen J.; Silva R. L. E.; Chu Z.; Sass N. C.; Lorenc V. E.; Green J. J.; Campochiaro P. A.; Popel A. S.; Pandey N. B. A collagen IV-derived peptide disrupts alpha5beta1 integrin and potentiates Ang2/Tie2 signaling. JCI Insight 2019, 4 ( (4), ). 10.1172/jci.insight.122043 PMC6478425 30668550

[ref13] Lu Y.; Li C.; Chen Q.; Liu P.; Guo Q.; Zhang Y.; Chen X.; Zhang Y.; Zhou W.; Liang D.; Zhang Y.; Sun T.; Lu W.; Jiang C. Microthrombus-Targeting Micelles for Neurovascular Remodeling and Enhanced Microcirculatory Perfusion in Acute Ischemic Stroke. Adv. Mater. 2019, 31 (21), e1808361 10.1002/adma.201808361. 30957932

[ref14] Wang Q.; Zhang Y.; Wu L.; Niu S.; Song C.; Zhang Z.; Lu G.; Qiao C.; Hu Y.; Yuen K.-Y.; Wang Q.; Zhou H.; Yan J.; Qi J. Structural and Functional Basis of SARS-CoV-2 Entry by Using Human ACE2. Cell 2020, 181 (4), 894–904. 10.1016/j.cell.2020.03.045. 32275855 PMC7144619

[ref15] Santos R. A. S.; Sampaio W. O.; Alzamora A. C.; Motta-Santos D.; Alenina N.; Bader M.; Campagnole-Santos M. J. The ACE2/Angiotensin-(1–7)/MAS Axis of the Renin-Angiotensin System: Focus on Angiotensin-(1–7). Physiol Rev. 2018, 98 (1), 505–553. 10.1152/physrev.00023.2016. 29351514 PMC7203574

[ref16] Mueller S. B.; Kontos C. D. Tie1: an orphan receptor provides context for angiopoietin-2/Tie2 signaling. J. Clin Invest 2016, 126 (9), 3188–3191. 10.1172/JCI89963. 27548526 PMC5004958

[ref17] Griffin M. E.; Sorum A. W.; Miller G. M.; Goddard W. A. 3rd; Hsieh-Wilson L. C. Sulfated glycans engage the Ang-Tie pathway to regulate vascular development. Nat. Chem. Biol. 2021, 17 (2), 178–186. 10.1038/s41589-020-00657-7. 33020664 PMC8087285

[ref18] Dang R.; Yang M.; Cui C.; Wang C.; Zhang W.; Geng C.; Han W.; Jiang P. Activation of angiotensin-converting enzyme 2/angiotensin (1–7)/mas receptor axis triggers autophagy and suppresses microglia proinflammatory polarization via forkhead box class O1 signaling. Aging Cell 2021, 20 (10), e13480 10.1111/acel.13480. 34529881 PMC8520723

[ref19] Pan R.-Y.; Ma J.; Kong X.-X.; Wang X.-F.; Li S.-S.; Qi X.-L.; Yan Y.-H.; Cheng J.; Liu Q.; Jin W.; Tan C.-H.; Yuan Z. Sodium rutin ameliorates Alzheimerâ€s diseaseâ€“like pathology by enhancing microglial amyloid-Î^2^ clearance. Science Advances 2019, 5 (2), eaau6328 10.1126/sciadv.aau6328. 30820451 PMC6393001

[ref20] Ye M.; Luo G.; Ye D.; She M.; Sun N.; Lu Y. J.; Zheng J. Network pharmacology, molecular docking integrated surface plasmon resonance technology reveals the mechanism of Toujie Quwen Granules against coronavirus disease 2019 pneumonia. Phytomedicine 2021, 85, 153401 10.1016/j.phymed.2020.153401. 33191068 PMC7837196

[ref21] Huang Y. F.; Bai C.; He F.; Xie Y.; Zhou H. Review on the potential action mechanisms of Chinese medicines in treating Coronavirus Disease 2019 (COVID-19). Pharmacol. Res. 2020, 158, 104939 10.1016/j.phrs.2020.104939. 32445956 PMC7239792

[ref22] Cerrato C. P.; Pirisinu M.; Vlachos E. N.; Langel U. Novel cell-penetrating peptide targeting mitochondria. FASEB J. 2015, 29 (11), 4589–4599. 10.1096/fj.14-269225. 26195590

[ref23] Sawada R.; Nakano-Doi A.; Matsuyama T.; Nakagomi N.; Takayuki Nakagomi T. CD44 expression in stem cells and niche microglia/macrophages following ischemic stroke. Stem Cell Investig 2020, 7, 4 10.21037/sci.2020.02.02. PMC7154319 32309418

[ref24] Tian C.; Asghar S.; Hu Z.; Qiu Y.; Zhang J.; Shao F.; Xiao Y. Understanding the cellular uptake and biodistribution of a dual-targeting carrier based on redox-sensitive hyaluronic acid-ss-curcumin micelles for treating brain glioma. Int. J. Biol. Macromol. 2019, 136, 143–153. 10.1016/j.ijbiomac.2019.06.060. 31199976

[ref25] Szeto H. H. Mitochondria-targeted cytoprotective peptides for ischemia-reperfusion injury. Antioxid Redox Signal 2008, 10 (3), 601–619. 10.1089/ars.2007.1892. 17999629

[ref26] Zhu Y.; Wang H.; Fang J.; Dai W.; Zhou J.; Wang X.; Zhou M. SS-31 Provides Neuroprotection by Reversing Mitochondrial Dysfunction after Traumatic Brain Injury. Oxid Med. Cell Longev 2018, 2018, 4783602 10.1155/2018/4783602. 30224944 PMC6129854

[ref27] Cerrato C. P.; Pirisinu M.; Vlachos E. N.; Langel Ã. Novel cell-penetrating peptide targeting mitochondria. FASEB J. 2015, 29 (11), 4589–4599. 10.1096/fj.14-269225. 26195590

[ref28] Mitchell W.; Ng E. A.; Tamucci J. D.; Boyd K. J.; Sathappa M.; Coscia A.; Pan M.; Han X.; Eddy N. A.; May E. R.; et al. The mitochondria-targeted peptide SS-31 binds lipid bilayers and modulates surface electrostatics as a key component of its mechanism of action. J. Biol. Chem. 2020, 295 (21), 7452–7469. 10.1074/jbc.RA119.012094. 32273339 PMC7247319

[ref29] Chavez J. D.; Tang X.; Campbell M. D.; Reyes G.; Kramer P. A.; Stuppard R.; Keller A.; Zhang H.; Rabinovitch P. S.; Marcinek D. J.; et al. Mitochondrial protein interaction landscape of SS-31. Proc. Natl. Acad. Sci. U. S. A. 2020, 117 (26), 15363–15373. 10.1073/pnas.2002250117. 32554501 PMC7334473

[ref30] Katarzyna Greda A.; Nowicka D. Hyaluronidase inhibition accelerates functional recovery from stroke in the mouse brain. J. Neurochem 2021, 157 (3), 781–801. 10.1111/jnc.15279. 33345310

[ref31] Barzegar M.; Stokes K. Y.; Chernyshev O.; Kelley R. E.; Alexander J. S. The Role of the ACE2/MasR Axis in Ischemic Stroke: New Insights for Therapy. Biomedicines 2021, 9, 1667 10.3390/biomedicines9111667. 34829896 PMC8615891

[ref32] Oboh G.; Adebayo A. A.; Ademosun A. O.; Olowokere O. G. Rutin restores neurobehavioral deficits via alterations in cadmium bioavailability in the brain of rats exposed to cadmium. Neurotoxicology 2020, 77, 12–19. 10.1016/j.neuro.2019.12.008. 31836556

[ref33] Abdel-Naby D. H.; Deghiedy N. M.; Rashed R. R.; El-Ghazaly M. A. Tailoring of chitosan/diacrylated pluronic system as a versatile nanoplatform for the amelioration of radiation-induced cognitive dysfunction. Int. J. Biol. Macromol. 2021, 193 (Pt B), 1507–1521. 10.1016/j.ijbiomac.2021.10.214. 34740686

[ref34] Wegner S.; Uhlemann R.; Boujon V.; Ersoy B.; Endres M.; Kronenberg G.; Gertz K. Endothelial Cell-Specific Transcriptome Reveals Signature of Chronic Stress Related to Worse Outcome After Mild Transient Brain Ischemia in Mice. Mol. Neurobiol 2020, 57 (3), 1446–1458. 10.1007/s12035-019-01822-3. 31758402 PMC7060977

[ref35] Wu C. C.; Wang L. C.; Su Y. T.; Wei W. Y.; Tsai K. J. Synthetic alpha5beta1 integrin ligand PHSRN is proangiogenic and neuroprotective in cerebral ischemic stroke. Biomaterials 2018, 185, 142–154. 10.1016/j.biomaterials.2018.09.014. 30243150

[ref36] Poittevin M.; Bonnin P.; Pimpie C.; Riviere L.; Sebrie C.; Dohan A.; Pocard M.; Charriaut-Marlangue C.; Kubis N. Diabetic Microangiopathy: Impact of Impaired Cerebral Vasoreactivity and Delayed Angiogenesis After Permanent Middle Cerebral Artery Occlusion on Stroke Damage and Cerebral Repair in Mice. Diabetes 2015, 64 (3), 999–1010. 10.2337/db14-0759. 25288671

[ref37] Sui R.; Zang L.; Bai Y. Administration of troxerutin and cerebroprotein hydrolysate injection alleviates cerebral ischemia/reperfusion injury by down-regulating caspase molecules. Neuropsychiatr Dis Treat 2019, 15, 2345–2352. 10.2147/NDT.S213212. 31695379 PMC6707350

[ref38] Liu Y.; Xue X.; Zhang H.; Che X.; Luo J.; Wang P.; Xu J.; Xing Z.; Yuan L.; Liu Y.; et al. Neuronal-targeted TFEB rescues dysfunction of the autophagy-lysosomal pathway and alleviates ischemic injury in permanent cerebral ischemia. Autophagy 2019, 15 (3), 493–509. 10.1080/15548627.2018.1531196. 30304977 PMC6351122

[ref39] Fan Y.; Lu H.; Liang W.; Garcia-Barrio M. T.; Guo Y.; Zhang J.; Zhu T.; Hao Y.; Zhang J.; Chen Y. E. Endothelial TFEB (Transcription Factor EB) Positively Regulates Postischemic Angiogenesis. Circ. Res. 2018, 122 (7), 945–957. 10.1161/CIRCRESAHA.118.312672. 29467198 PMC5918429

[ref40] Doronzo G.; Astanina E.; Cora D.; Chiabotto G.; Comunanza V.; Noghero A.; Neri F.; Puliafito A.; Primo L.; Spampanato C.; Settembre C.; Ballabio A.; Camussi G.; Oliviero S.; Bussolino F. TFEB controls vascular development by regulating the proliferation of endothelial cells. EMBO J. 2019, 38 ( (3), ). 10.15252/embj.201798250 PMC6356157 30591554

[ref41] Lefere S.; Van de Velde F.; Hoorens A.; Raevens S.; Van Campenhout S.; Vandierendonck A.; Neyt S.; Vandeghinste B.; Vanhove C.; Debbaut C.; et al. Angiopoietin-2 Promotes Pathological Angiogenesis and Is a Therapeutic Target in Murine Nonalcoholic Fatty Liver Disease. Hepatology 2019, 69 (3), 1087–1104. 10.1002/hep.30294. 30259536

[ref42] Liu C.; Liu S.; Xiong L.; Zhang L.; Li X.; Cao X.; Xue J.; Li L.; Huang C.; Huang Z. Genistein-3′-sodium sulfonate Attenuates Neuroinflammation in Stroke Rats by Down-Regulating Microglial M1 Polarization through alpha7nAChR-NF-kappaB Signaling Pathway. Int. J. Biol. Sci. 2021, 17 (4), 1088–1100. 10.7150/ijbs.56800. 33867831 PMC8040300

[ref43] Xiong X. Y.; Liu L.; Yang Q. W. Functions and mechanisms of microglia/macrophages in neuroinflammation and neurogenesis after stroke. Prog. Neurobiol 2016, 142, 23–44. 10.1016/j.pneurobio.2016.05.001. 27166859

[ref44] Patel N. Acute Oral Toxicity and Anti Tussive Effect of Kashloff Syrup (Poly Herbal Formulation) on So2 Induced Cough Model. Journal of Medical Pharmaceutical And Allied Sciences 2019, 8 (5), 2333–2337. 10.22270/jmpas.v8i5.865.

[ref45] van der Spek D.; van Arendonk J. A.; Bovenhuis H. Genome-wide association study for claw disorders and trimming status in dairy cattle. J. Dairy Sci. 2015, 98 (2), 1286–1295. 10.3168/jds.2014-8302. 25497826

[ref46] Chang J. Y.; Kim W. J.; Kwon J. H.; Kim B. J.; Kim J. T.; Lee J.; Cha J. K.; Kim D. H.; Cho Y. J.; Hong K. S.; et al. Prestroke Glucose Control and Functional Outcome in Patients With Acute Large Vessel Occlusive Stroke and Diabetes After Thrombectomy. Diabetes Care 2021, 44 (9), 2140–2148. 10.2337/dc21-0271. 34215632 PMC8740925

[ref47] Bright C. J.; Hawkins M. M.; Guha J.; Henson K. E.; Winter D. L.; Kelly J. S.; Feltbower R. G.; Hall M.; Cutter D. J.; Edgar A. B.; et al. Risk of Cerebrovascular Events in 178â€‰962 Five-Year Survivors of Cancer Diagnosed at 15 to 39 Years of Age. Circulation 2017, 135 (13), 1194–1210. 10.1161/CIRCULATIONAHA.116.025778. 28122884 PMC7614827

[ref48] Sung Y. C.; Jin P. R.; Chu L. A.; Hsu F. F.; Wang M. R.; Chang C. C.; Chiou S. J.; Qiu J. T.; Gao D. Y.; Lin C. C.; et al. Delivery of nitric oxide with a nanocarrier promotes tumour vessel normalization and potentiates anti-cancer therapies. Nat. Nanotechnol 2019, 14 (12), 1160–1169. 10.1038/s41565-019-0570-3. 31740794

