# Supplementary material for: A Triple-Targeted Rutin-Based Self-Assembled Delivery Vector for Treating Ischemic Stroke by Vascular Normalization and Anti-Inflammation via ACE2/Ang1-7 Signaling
Source: ACS Cent Sci. 2023 Jun 5;9(6):1180–99. doi: 10.1021/acscentsci.3c00377 (PMC10311651; doi:10.1021/acscentsci.3c00377)
Supplement: Supplementary file 1 — oc3c00377_si_001.pdf [file oc3c00377_si_001.pdf]

Supporting Information

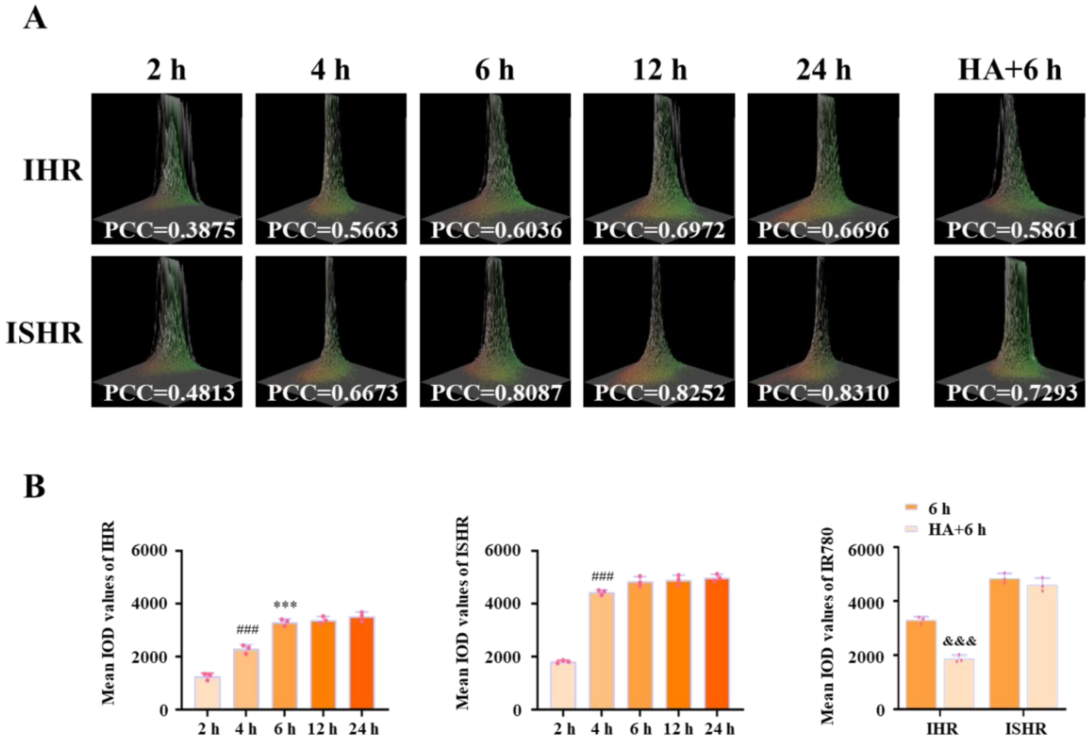

Fig. S1. Subcellular co-localization analyses in Fig. 2G-I. (A) Pearson’s correlation coefficient (PCC). (B) Fluorescence semi quantitative analysis in Fig. 2G-I.

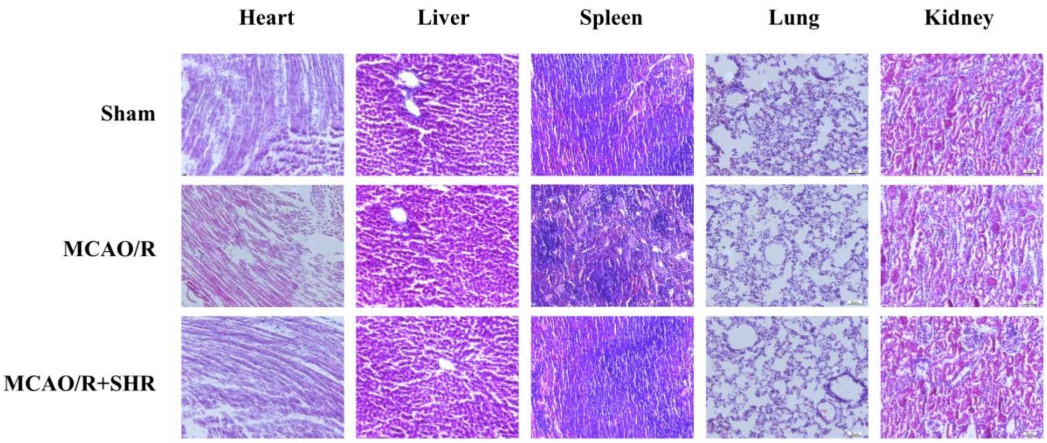

Figure S2. HE staining of heart, liver, spleen, lung and kidney in rats 21 days after cerebral ischemia reperfusion.

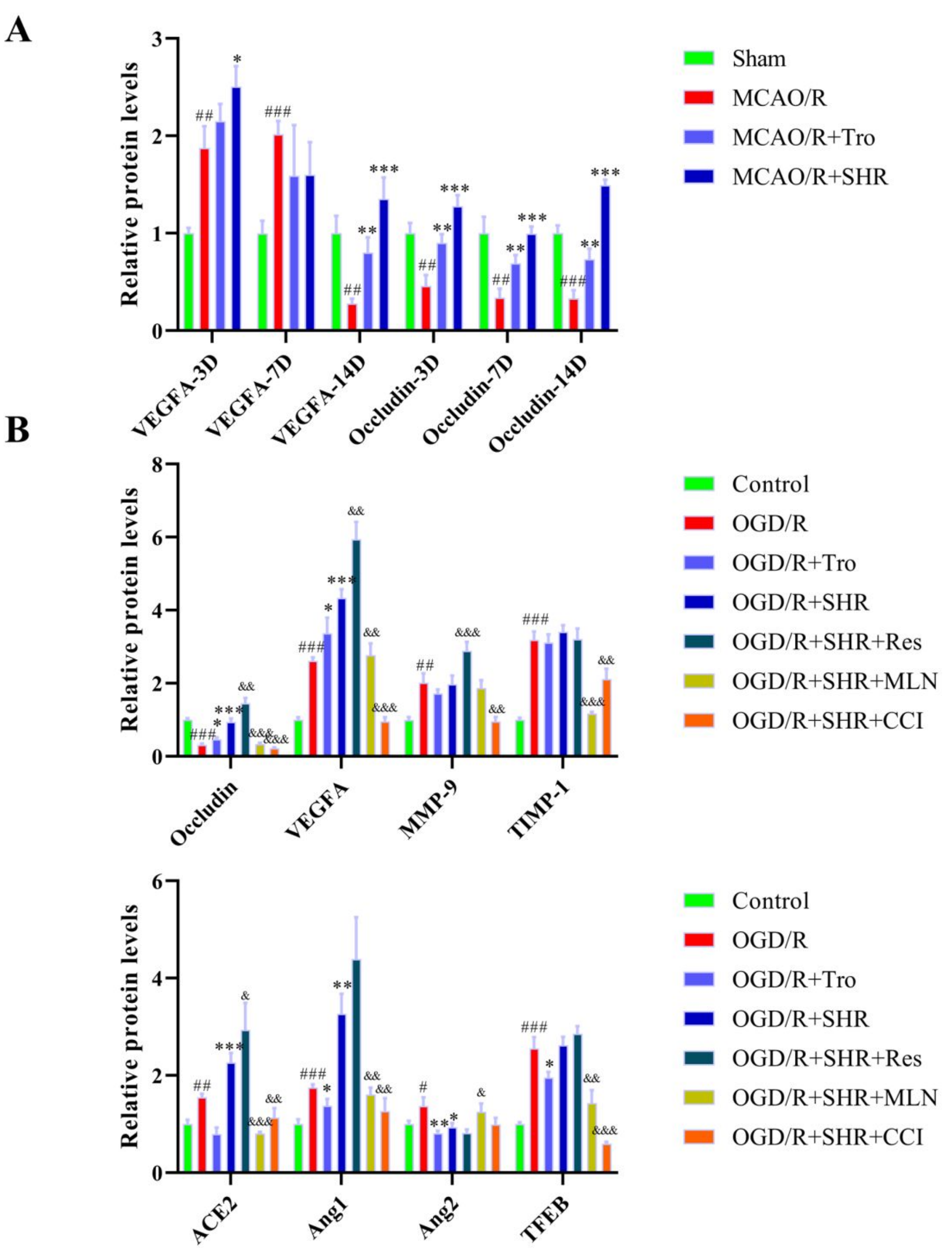

Fig. S3. Quantitative analysis of protein expression in Fig. 5. (A) Quantitative analysis of protein expression in Fig. 5D. (B) Quantitative analysis of protein expression in Fig. 5I.

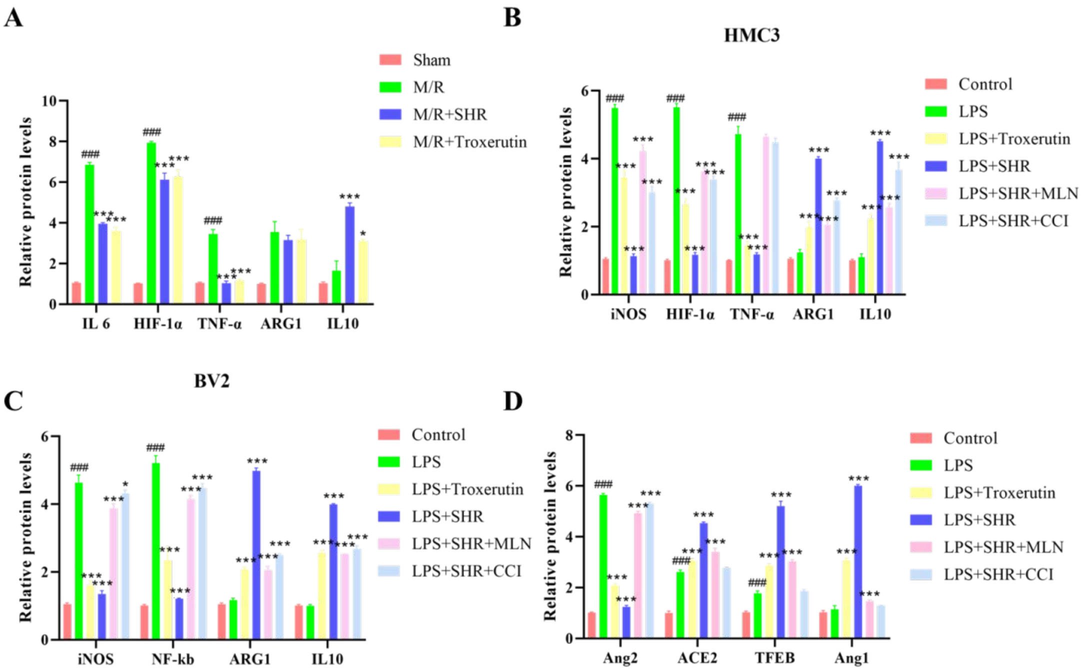

Fig. S4. Quantitative analysis of protein expression in Fig. 6. (A) Quantitative analysis of protein expression in Fig. 6D. (B) Quantitative analysis of protein expression in Fig. 6G. (A) Quantitative analysis of protein expression in Fig. 6G. (B) Quantitative analysis of protein expression in Fig. 6I.

Table S1: Calculation of LD_{50} for SHR micelles.

Dose (mg/kg)	Dose logarithm	1	2	3	4	5	6	7	8	9
60	1.7782							×		×
55	1.7404				×		√		√	
50	1.6990			√		√				
30	1.4771		√							
20	1.3010	√								

LD_{50} =56.25 mg/kg
